# Supplementary material for: Mechanism of effector capture and delivery by the type IV secretion system from Legionella pneumophila
Source: Nat Commun. 2020 Jun 8;11:2864. doi: 10.1038/s41467-020-16681-z (PMC7280309; doi:10.1038/s41467-020-16681-z)
Supplement: Supplementary file 1 — Supplementary Information [file 41467_2020_16681_MOESM1_ESM.pdf]

SUPPLEMENTARY INFORMATION

Mechanism of effector capture and delivery by the type IV secretion system from *Legionella pneumophila*

Amit Meir[1,4,5*], Kevin Macé[1,4], Natalya Lukoyanova[1], David Chetrit[2], Manuela K. Hospenthal[1,6], Adam Redzej[1], Craig Roy[2*], and Gabriel Waksman[1,3*]

[1] Institute of Structural and Molecular Biology, Birkbeck and UCL, Malet Street, London WC1E 7HX, UK; [2] Boyer Center for Molecular Medicine, Department of Microbial Pathogenesis, Yale University, 295 Congress Avenue, New Haven, CT 06536-0812, USA; [3]Institute of Structural and Molecular Biology, University College London, Gower Street, London WC1E 6BT, UK. [4] These authors contributed equally to this work. [5] Present address: Boyer Center for Molecular Medicine, Department of Microbial Pathogenesis, Yale University, 295 Congress Avenue, New Haven, CT 06536-0812, USA. [6] Present address: Institute of Molecular Biology and Biophysics, Department of Biology, ETH Zürich, Otto-Stern-Weg 5, 8093 Zürich, Switzerland

Supplementary Table 1. Strains used in this study

Strain	Description	Source/ Reference
E.coli K12 strains used in this study		
E. coli Mach1	Δ*recA1398 endA1 fhuA* Φ80Δ(*lac*)M15 *Δ(*lac*)X74* hsdR*(*r$_K^-$m$_K^+$*)*	Invitrogen
E. coli DH5α λpir	sup E44, ΔlacU169 (ΦlacZΔM15), recA1, endA1, hsdR17, thi-1, gyrA96, relA1, λpir phage lysogen	Zuckman 1999 [1]
E. coli CR019	MT607 *E. coli* containing plasmid pRK600; ColE1 replicon with RK2 transfer genes, Cm^R	Hubber 2014 [2]
Legionella pneumophila strains used in this study		
Lp01	Strep^R, *Legionella pneumophila* serogroup 1, Lp01 *rpsL*	Berger and Isberg 1993 [3]
Lp02	Strep^R, *Legionella pneumophila* serogroup 1, Lp01 *rpsL SmR, r–thyA*	Berger and Isberg 1993 [3]
Lp01 DotL$_{strep}$	Strep^R, Lp01 with a Strep tag at the C-terminus of DotL	This study
Lp02 DotL$_{strep}$	Strep^R, Lp02 with a Strep tag at the C-terminus of DotL	This study
ΔT4SS	Strep^R, Lp01 chromosomal deletions of three loci: *icmX–dotA*, *dotB–dotD*, and *icmT–dotU*	Kubori 2014 [4]
Δ*dotY*	Strep^R, Lp01 *lpg0294* chromosomal deletion	This study
Δ*dotz*	Strep^R, Lp01 *lpg1549* chromosomal deletion	This study
Δ*dotYdotZ*	Strep^R, Lp01 *lpg0294 and lpg1549* chromosomal deletion	This study
Δ*dotY* +p*dotY*	Strep^R, CM^R, Δ*dotY* + pDotY	This study
Δ*dotZ* +p*dotZ*	Strep^R, CM^R, Δ*dotZ* + pDotZ	This study
Δ*dotB*	Strep^R, Lp01 *dotB* chromosomal deletion	This study
M1	Strep^R, Lp01 Δ*dotB dotM* T205R, Y211R, L208R	This study
M3	Strep^R, Lp01 *dotN* 200-end deletion	This study
M4	Strep^R, Lp01 *dotZ* 283-end deletion	This study
M5	Strep^R, Lp01 Δ*dotB dotM* Q326R, T327R	This study
M6	Strep^R, Lp01 Δ*dotB dotL* A363R, E364R, D366R	This study
M7	Strep^R, Lp01 Δ*dotB dotM* V300R, P302R, S303R	This study
Δ*dotB* p*dotB*	Strep^R, CM^R, Δ*dotB* + pDotB$_{strep}$	This study
M1 p*dotB*	Strep^R, CM^R, M1 + pDotB$_{strep}$	This study
M5 p*dotB*	Strep^R, CM^R, M5 + pDotB$_{strep}$	This study
M6 p*dotB*	Strep^R, CM^R, M6 + pDotB$_{strep}$	This study
M7 p*dotB*	Strep^R, CM^R, M7 + pDotB$_{strep}$	This study
Lp01 CegC3$_{FL}$	Strep^R, CM^R, Lp01 + pCYA-CegC3$_{FL}$	Meir 2018 [6]
Lp01 Lpg1663$_{FL}$	Strep^R, CM^R, Lp01 + pCYA-Lpg1663$_{FL}$	This study
Lp01 RalF$_{FL}$	Strep^R, CM^R, Lp01 + pCYA-RalF$_{FL}$	Nagai 2005 [5]
Lp01 LegC8$_{FL}$	Strep^R, CM^R, Lp01 + pCYA-LegC8$_{FL}$	Meir 2018 [6]
Lp01 Lem21$_{FL}$	Strep^R, CM^R, Lp01 + pCYA-Lem21$_{FL}$	This study
Lp01 Cya-dotY	Strep^R, CM^R, Lp01 + pCYA-DotY$_{FL}$	This study
Lp01 Cya-dotZ	Strep^R, CM^R, Lp01 + pCYA-DotZ$_{FL}$	This study

ΔT4SS $CegC3_{FL}$	$Strep^R$, CM^R, ΔT4BS + pCYA-$CegC3_{FL}$	Meir 2018 [6]
ΔT4SS $Lpg1663_{FL}$	$Strep^R$, CM^R, ΔT4BS + pCYA-$Lpg1663_{FL}$	This study
ΔT4SS $RalF_{FL}$	$Strep^R$, CM^R, ΔT4BS + pCYA-$RalF_{FL}$	This study
ΔT4SS $LegC8_{FL}$	$Strep^R$, CM^R, ΔT4BS + pCYA-$LegC8_{FL}$	Meir 2018 [6]
ΔT4SS $Lem21_{FL}$	$Strep^R$, CM^R, ΔT4BS + pCYA-$Lem21_{FL}$	This study
ΔT4SS Cya-DotY	$Strep^R$, CM^R, ΔT4BS + pCYA-$DotY_{FL}$	This study
ΔT4SS Cya- DotZ	$Strep^R$, CM^R, Δ*dotY* + pCYA-$DotZ_{FL}$	This study
Δ*dotY* $CegC3_{FL}$	$Strep^R$, CM^R, Δ*dotY* + pCYA-$CegC3_{FL}$	This study
Δ*dotY* $Lpg1663_{FL}$	$Strep^R$, CM^R, Δ*dotY* + pCYA-$Lpg1663_{FL}$	This study
Δ*dotY* $RalF_{FL}$	$Strep^R$, CM^R, Δ*dotY* + pCYA-$RalF_{FL}$	This study
Δ*dotY* $LegC8_{FL}$	$Strep^R$, CM^R, Δ*dotY* + pCYA-$LegC8_{FL}$	This study
Δ*dotY* $Lem21_{FL}$	$Strep^R$, CM^R, Δ*dotY* + pCYA-$Lem21_{FL}$	This study
Δ*dotZ* $CegC3_{FL}$	$Strep^R$, CM^R, Δ*dotZ* + pCYA-$CegC3_{FL}$	This study
Δ*dotZ* $Lpg1663_{FL}$	$Strep^R$, CM^R, Δ*dotZ* + pCYA-$Lpg1663_{FL}$	This study
Δ*dotZ* $RalF_{FL}$	$Strep^R$, CM^R, Δ*dotZ* + pCYA-$RalF_{FL}$	This study
Δ*dotZ* $LegC8_{FL}$	$Strep^R$, CM^R, Δ*dotZ* + pCYA-$LegC8_{FL}$	This study
Δ*dotZ* $Lem21_{FL}$	$Strep^R$, CM^R, Δ*dotZ* + pCYA-$Lem21_{FL}$	This study
Δ*dotYdotZ* $CegC3_{FL}$	$Strep^R$, CM^R, Δ*dotYdotZ* + pCYA-$CegC3_{FL}$	This study
Δ*dotYdotZ* $Lpg1663_{FL}$	$Strep^R$, CM^R, Δ*dotYdotZ* + pCYA-$Lpg1663_{FL}$	This study
Δ*dotYdotZ* $RalF_{FL}$	$Strep^R$, CM^R, Δ*dotYdotZ* + pCYA-$RalF_{FL}$	This study
Δ*dotYdotZ* $LegC8_{FL}$	$Strep^R$, CM^R, Δ*dotYdotZ* + pCYA-$LegC8_{FL}$	This study
Δ*dotYdotZ* $Lem21_{FL}$	$Strep^R$, CM^R, Δ*dotYdotZ* + pCYA-$Lem21_{FL}$	This study
Eukaryotic cell lines used in this study		
CHO FcγRII	Chinese hamster ovary expressing FcγRII	Nagai 2005 [5]
Acanthamoeba castellanii	ATCC 30234	

Supplementary Table 2. Plasmids used in this study

Construct	Description and Reference	Primers<br>Forward	 Reverse
pSR47S	Km^R; (J.J Merriam 1997 [7])		
pSR47S-Lin	Linearize pSR47S with In-Fusion to yield an open vector	p47SF	p47SR
pSR47S-LI	Km^R; Full length DotL from *L. pneumophila* with 1000bp upstream and downstream cloned into pSR47S-Lin backbone (this study)	p47060F	p47061R
pSR47S-MI	Km^R; Full length DotM from *L. pneumophila* with 1000bp upstream and downstream cloned into pSR47S-Lin backbone (Meir 2018 [6])		
pSR47S-NI	Km^R; Full length DotN from *L. pneumophila* with 1000bp upstream and downstream cloned into pSR47S-Lin backbone (this study)	p47035F	p47036R
pSR47S-YI	Km^R; Full length DotY (lpg0294) from *L. pneumophila* with 1000bp upstream and downstream cloned into pSR47S-Lin backbone (this study)	p47023F	p47024R
pSR47S-ZI	Km^R; Full length DotZ (lpg1549) from *L. pneumophila* with 1000bp upstream and downstream cloned into pSR47S-Lin backbone (this study)	p47019F	p47020R
pSR47S-LI_{strep}	Km^R; pSR47S-LI derivative encoding a strep tag on the C-terminal of DotL (this study)	p47014F	P47014StR
pSR47S-$DotMI_{M1}$	Km^R; pSR47S-MI derivative encoding a triple T205R, Y211R, L208R mutation (this study)	p47041inF	p47042inR
pSR47S-$DotNI_{M3}$	Km^R; pSR47S-NI derivative encoding a deletion of residues 200-214 (this study)	p47050F	p47049R
pSR47S-$DotZI_{M4}$	Km^R; pSR47S-ZI derivative encoding a deletion of residues 283-294 (this study)	p47022F	p47051R
pSR47S-$DotM_{M5}$	Km^R; pSR47S-MI derivative encoding a double Q326R, T327R mutation (this study)	p47052inF	p47053inR
pSR47S-$DotLI_{M6}$	Km^R; pSR47S-LI derivative encoding a triple mutation A363R, E364R, D366R (this study)	p47054inF	p47055inR
pSR47S-$DotMI_{M7}$	Km^R; pSR47S-MI derivative encoding a triple mutation V300R, P302R, S303R (this study)	p47056inF	p47057inR
pSR47S-Δ*dotY*	Km^R; pSR47S-YI derivative with *dotY* deletion (this study)	p47026F	p47025R
pSR47S-Δ*dotZ*	Km^R; pSR47S-ZI derivative with *dotZ* deletion (this study)	p47022F	p47021R
pJB1806	Cm^R Amp^R; (Bardill 2005 [10])		
pJB1806-Lin	pJB1806 without its lac promoter, linearized with In-Fusion to yield an open vector (this study)	pJB002F	pJB001R
p*dotY*	Cm^R; *dotY* with 200bp upstream and downstream cloned into pJB1806-Lin backbone (this study)	pJB005F	pJB006R
p*dotZ*	Cm^R; *dotZ* with 200bp upstream and	pJB003F	pJB004R

	downstream cloned into pJB1806-Lin backbone (this study)		
pSR47S-BI	Km^R; Full length DotB from *L. pneumophila* with 1000bp upstream and downstream cloned into pSR47S-Lin backbone (this study)	p47066F	p47067R
pSR47S-Δ*dotB*	Km^R; pSR47S-BI derivative with *dotB* deletion (this study)	p47068F	p47069R
pCYA	Cm^R;Cya domain cloned into pMMB207 (ATCC®37809) (Nagai 2005)		
pCYA-Lin	Linearize pCYA with In-Fusion to yield an open vector	CYA001F	CYA001R
pCYA-$CegC3_{FL}$	Cm^R; Linearize pCya with full length CegC3 (lpg1144) from L. pneumophila (Meir 2018 [6])		
pCYA-$Lpg1663_{FL}$	Cm^R; Linearize pCya with full length Lpg1663 from *L. pneumophila* (this study)	CYA013F	CYA012R
pCYA-RalF	Cm^R; Linearize pCya with Full length RalF (lpg1950) from *L. pneumophila* (Nagai 2005)		
pCYA-$LegC8_{FL}$	Cm^R; Linearize pCya with full length LegC8 (lpg2862) from *L. pneumophila* (Meir 2018 [6])		
pCYA-$Lem21_{FL}$	Cm^R; Linearize pCya with full length Lem21 (lpg2248) from *L. pneumophila* (this study)	CYA019F	CYA018R
pCYA-$DotY_{FL}$	Cm^R; Linearize pCya with full length DotY from *L. pneumophila* (this study)	CYA023F	CYA024R
pCYA-$DotZ_{FL}$	Cm^R; Linearize pCya with full length DotZ from *L. pneumophila* (this study)	CYA021F	CYA022R
pMMB207-Lin	Linearize p MMB207 (ATCC®37809) with In-Fusion to yield an open vector	CYA001F	MMB_LinR
pDotB	Cm^R;DotB with C-terminus Strep tag cloned into pMMB207-Lin (this study)	MMB017F	MMB018R

Supplementary Table 3. Primers used in this study

Primer name	Primer sequence 5'-3'
p47SF	ggatcccccgggctgcaggaattcg
p47SR	ccactagttctagagcggccgcc
p47014F	agcgcttggagccacccgcagttcgaaaaataaagactcagtcggcaattggaggtagaag
p47014StR	gtggctccaagcgcttgttaattcctctgcagccttttattggc
p47019F	ctctagaactagtgggagggtataatccacatgtggctaaagaataatg
p47020R	cagcccgggggatcccagccatacttgaccaattatttcagctgttg
p47021R	gactcaattcatcatctttttagatctcgtccat
p47022F	gatgatgaattgagtccattatactttgatagaacgattggagagcg
p47023F	ctctagaactagtgggtcatccgtttattcgtgatgaattctacgcc
p47024R	cagcccgggggatccccgattttatccgggcctttgatctatgtgg
p47025R	catctcttgtgggcagtgtttattttggcat
p47026F	ctgcccacaagagatgaaattgaatccggtgctgaagctccaacc
p47035F	ctctagaactagtgggatgtcgagtaatacgagtatcaaagagcc
p47036R	cagcccgggggatcccagggcacatgtgaaggaagaatacgag
p47041inF	atgcaacgtggtcctcgttgggatggttttgaacgttgctctcc
p47042inR	gaggaccacgttgcatacgaaatactcgtttggcatcg
p47049R	gtacttttactgtttagaattttgccctagagggtaatagcc
p47050F	taaacagtaaaagtactattaataattgaggacaaagg
p47051R	gactcaattcatcatcttatcggttaatcgcatcttgtgcaggg
p47052inF	cgtcgtccatactctgaagttgcaggtcctttcg
p47053inR	ggagtatggacgacgcctgcctacacaattcaacatataccataaacg
p47054inF	cgtcgtgtccgttttaaagatgtggtactcaacagacgtatattag
p47055inR	aaaacggacacgacgtaaattggtacgaataatatgaccataagtatctg
p47056inF	cgtgttcgtcgttcagagtttttatggctcaaaccagtcgac
p47057inR	ctgaacgacgaacacgaccatcttctcgtgctgcttcaagcaaagaagc
p47060F	taaagactcagtcggcaattggaggtagaag
p47061R	ccgactgagtctttatgttaattcctctgcagccttttattg
p47066F	ttttccttttgcggccgcaaagatgagagtctggccgagatcc
p47067R	ttttccttttccgcggataacaacgtcgatttcatcaccaagg
p47068F	acctattatagcaccagtaagttaatgtcttatcaacaataatcaaaattaatctg
p47069R	aaacagattaattttgattattgttgataagacattaacttactggtgctataatagg
pJB001R	caagcttggtaagaggttccaactttcacc
pJB002F	caacgcaattaatgtaagttagcgcgaattg
pJB003F	ggtgaaagttggaacctcttaccaagcttgcacaatgagcatgatcgagcaataaaagag
pJB004R	caattcgcgctaacttacattaattgcgttgctatcacagactttgctacagcatcagg
pJB005F	ggtgaaagttggaacctcttaccaagcttggtggatgaatttaccttcgactccatgaag
pJB006R	caattcgcgctaacttacattaattgcgttgccaaaggtttttgccaactgcagacaag
CYA001F	ctgcaggcatgcaagcttggctgttttg
CYA001R	cgcgccaccgccgctgtcatagccggaatcctggcgttcc
CYA012R	cttgcatgcctgcagttaaactgaggccgagtttcttgactcttcatc
CYA013F	agcggcggtggcgcgatgattatgtttgagtcttttcgaaaatgtttgtctgc
CYA018R	cttgcatgcctgcagttaaggaacatatctgttggcaaacaattgcacttc
CYA019F	agcggcggtggcgcgatggcgaagacaattaaggctactggag
CYA021F	agcggcggtggcgcgatggacgagatcaaaaaagatgatgaattgagtc
CYA022R	cttgcatgcctgcagttagcgctctccaatcgttctatcaaag
CYA023F	agcggcggtggcgcgatgccaaaatacacactgcccacaagagatg
CYA024R	cttgcatgcctgcagctatctgatggattgagtggttggagcttc
MMB_LinR	catggatccccgggtaccgagctcgaattctgtttc

MMB017F	acccggggatccatggcaacctattatagcaccagtaagttaatg
MMB018R	cttgcatgcctgcagttatttttcgaactgcgggtggctccaagcgctttgttgatattctttggccccag caatg

Supplementary Table 4. Cryo-EM data collection, refinement and validation statistics

	DotLMNYZ EMBD 10350 PDB 6SZ9	DotL	DotM	DotN	DotY	DotZ
**Data collection and processing**						
Magnification	130,000					
Voltage (kV)	300					
Electron exposure ($e^{-}/Å^{2}$)	54					
Defocus range (μm)	-1.5 to -3.5					
Pixel size (Å)	1.045					
Symmetry imposed	C1					
Initial particle images (no.)	626,230					
Final particle images (no.)	219,593					
Map resolution (Å)	3.73					
FSC threshold	0.143					
Map resolution range (Å)	3.2-7.0					
Map sharpening B factor ($Å^{2}$)	-86.26					
**Refinement**[1]						
Initial model used (PDB code)	1GKI, 5X42, 6EXD	5X42	6EXD	5X42	*de novo*	*de novo*
Model resolution (Å)	3.9	4.0	3.9	3.9	4.1	3.9
FSC threshold	0.50	0.50	0.50	0.50	0.50	0.50
CC Model vs. Data (mask)	0.79	0.77	0.79	0.79	0.76	0.79
Model composition						
Nonhydrogen atoms	10,233	3,665	2,085	1,616	583	2.284
Protein residues	1,290	473	259	202	73	283
Ligands	1	0	0	1	0	0
B factors ($Å^{2}$)						
Protein	58.3 (124 – 16)	73.1 (121 – 21)	58.6 (100 – 33)	53.79 (83 – 33)	77.7 (103 – 50)	51.2 (103 – 19)
Ligand	70.3	---	---	70.3	---	---
R.m.s. deviations						
Bond lengths (Å)	0.004	0.007	0.005	0.005	0.008	0.010
Bond angles (°)	0.803	0.917	0.818	0.740	0.933	1.102
**Validation**[1]						
MolProbity score	1.51	1.70	1.22	1.17	1.65	1.74
Clashscore	5.79	5.96	3.59	2.82	4.23	5.64
Rotamer outliers (%)	0.27	0.25	0.45	0.00	0.00	0.78
Ramachandran plot						
Favored (%)	96.86	94.62	97.67	97.50	92.96	93.24
Allowed (%)	3.14	5.38	2.33	2.50	7.04	6.76
Disallowed (%)	0.00	0.00	0.00	0.00	0.00	0.00

[1.] Determined by phenix.mtriage and phenix.molprobity (values in parenthesis denote highest and lowest values).

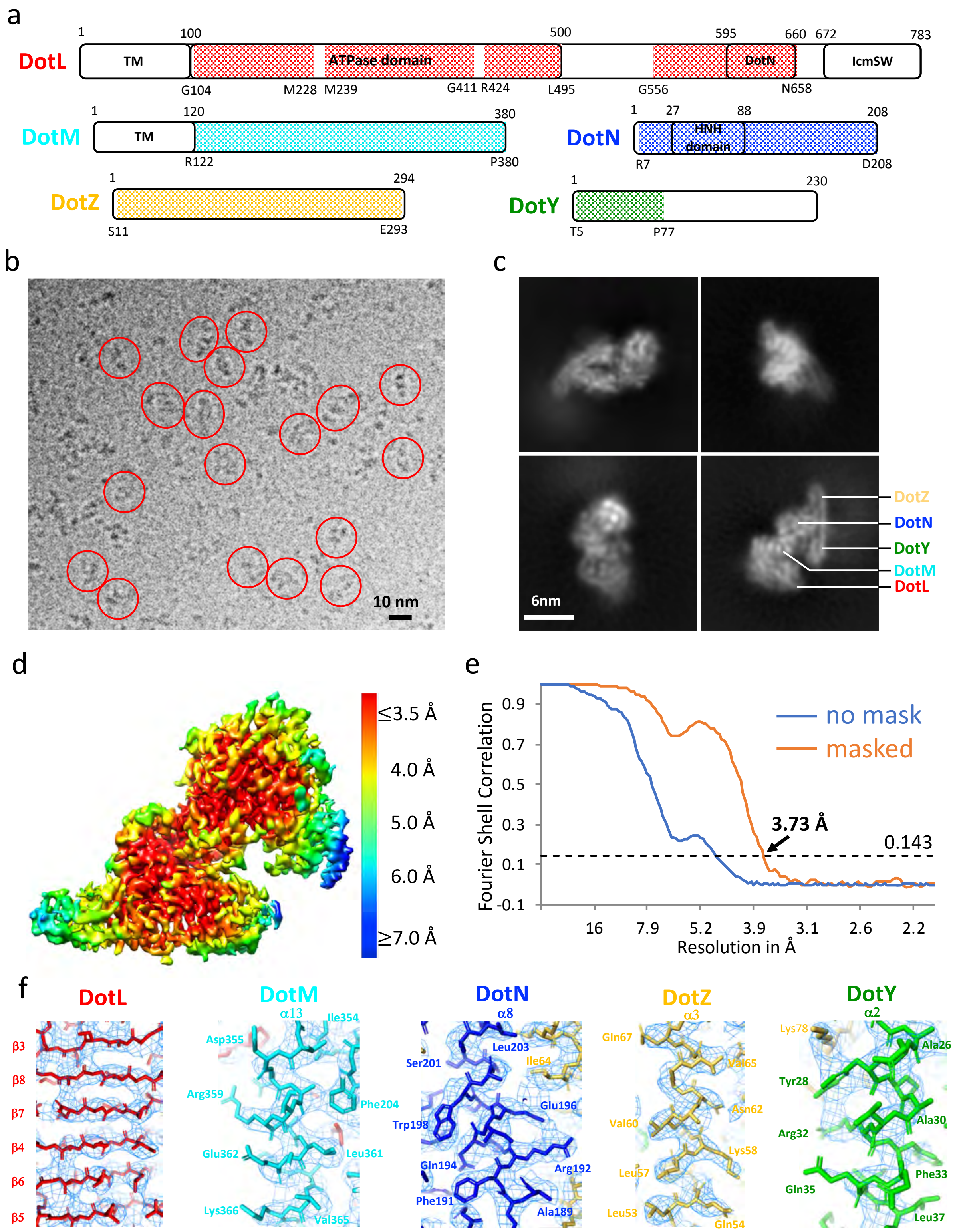

Supplementary Figure 1. Primary structure of DotL, DotM, DotN, DotY and DotZ, and structure determination of the DotLMNYZ hetero-pentameric complex. a, Primary structure of the T4CC complex components. For DotL, DotM, and DotN, prior knowledge of the domain structure of the proteins is shown together with boundary residues and domain names. Colored parts for DotL, DotM, DotN, DotZ and DotY (red, cyan, blue, orange yellow and green, respectively) show the parts of the proteins for which high resolution electron density could be observed in the hetero-pentameric T4CC core map and therefore for which

an atomic model could be built. **b**, Cryo-EM micrograph of the fully-assembled T4CC. Red circles and ovals indicate examples of particles. 19,491 such micrographs over five datasets were collected. **c**, Representative 2D class averages of the hetero-pentameric complex obtained using CRYOSPARC. 626,230 particles were used to generate these class averages (see Methods). Positions of proteins are indicated for one of the class averages. **d**, Local resolution variations of the T4CC map calculated using CRYOSPARC and coloured as indicated in scale below. The map was contoured at 6.5 σ level. **e**, Resolution of the T4CC complex core derived from Fourier Shell Correlation (FSC) between independently refined half-maps. The dotted line represents the 0.143 FSC cut-off, which indicates a nominal resolution of 3.73 Å. For the curve labelled "masked", the FSC was calculated using a soft-edge mask encompassing the hetero-pentameric unit. **f**, Representative regions of the electron density map visualized at 6.5 σ level with the final model of the T4CC hetero-pentameric core in stick representation color-coded per proteins as in Fig. 1e. The secondary structures and residues shown are labelled.

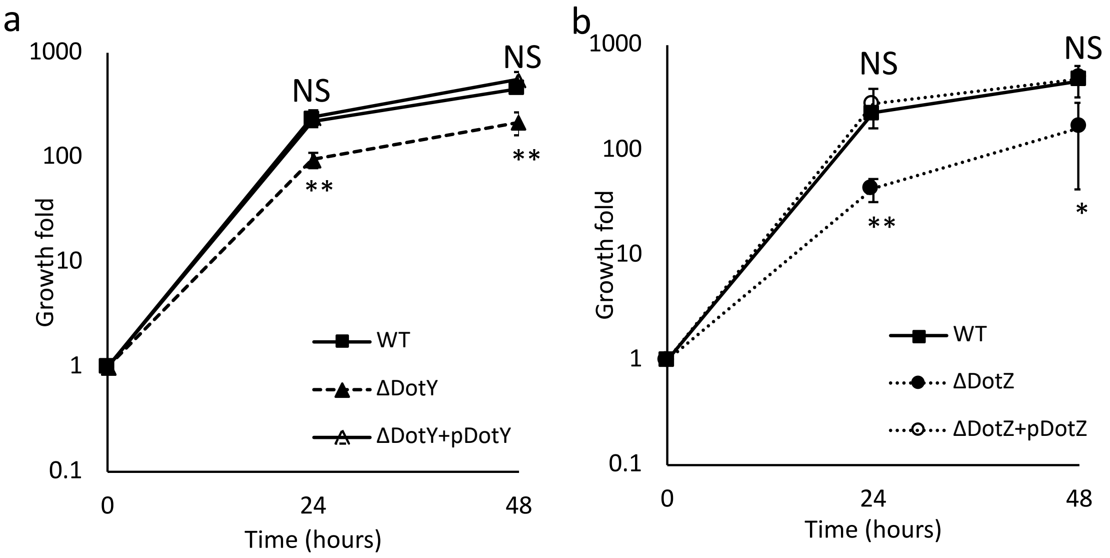

Supplementary Figure 2. Intracellular growth defect of Δ*dotY*, Δ*dotZ*, their complementations with wild-type genes in *A. castellanii*. **a**, Intracellular growth of Δ*dotY* mutant. Wild-type (Lp01, filled squares), Δ*dotY* (filled triangles), and Δ*dotY* with complementing wild-type *dotY* (p*dotY*, empty triangles). **b**, Intracellular growth of Δ*dotZ* mutant. Wild-type (Lp01, filled squares), Δ*dotZ* (filled circles), and Δ*dotZ* with complementing wild-type *dotZ* (p*dotZ*, empty circles). Data are representative of three independent experiments (n=3), each with biological triplicates. Graphs report mean intracellular growth fold ± standard deviation for each strain. P values of mutant strains in comparison to wild-type, calculated by two-tailed Student's t test. P values for Δ*dotY* mutant were 0.0004 and 0.0031 for 24 and 48 hours post infection, respectively. P values for Δ*dotZ* mutant were < 0.0001 and 0.0159 for 24 and 48 hours post infection, respectively, indicating a significant difference. For the complementing strains, P values were 0.36 and 0.15 for Δ*dotY*+p*dotY* for 24 and 48 hours post infection, respectively, and 0.505, 0.82 for Δ*dotZ*+p*dotZ* for 24 and 48 hours post infection, respectively. * and ** indicate P values < 0.05 and < 0.01, NS = Not significant (P>0.05). Source data are provided as a Source Data file.

a

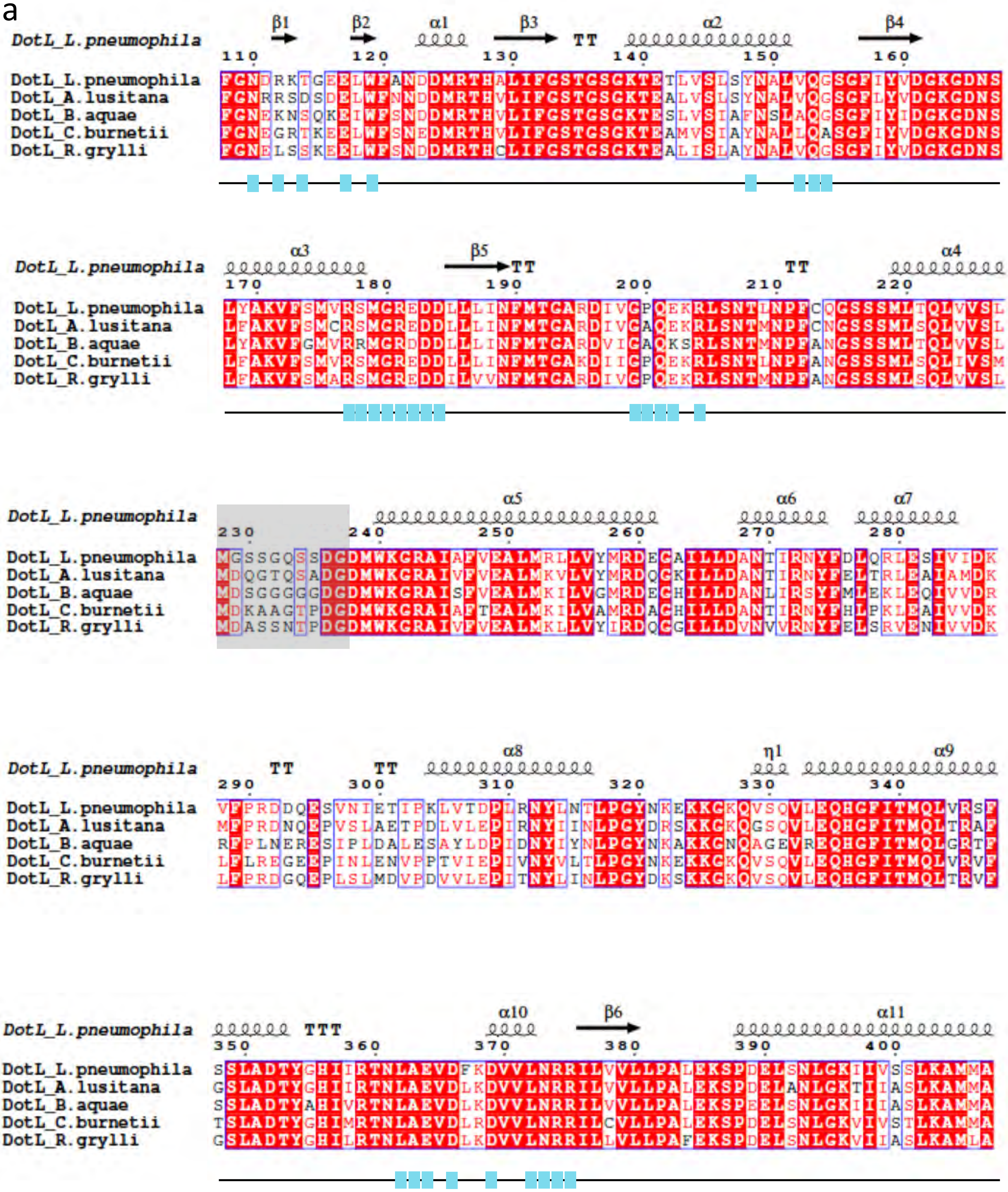
DotL_L.pneumophila
β1 β2 α1 β3 TT α2 β4
110 120 130 140 150 160
DotL_L.pneumophila FGNDRKTGEELWFANDDMRTHALIFGSTGSGKTETLVSLSYNALVQGSGFIYVDGKGDNS
DotL_A.lusitana FGNRRSDSDELWFNNDDMRTHVLIFGSTGSGKTEALVSLSYNALVQGSGFLYVDGKGDNS
DotL_B.aquae FGNEKNSQKEIWFSNDDMRTHVLIFGSTGSGKTESLVSIAFNSLAQGSGFIYIDGKGDNS
DotL_C.burnetii FGNEGRTKEELWFSNEDMRTHVLIFGSTGSGKTEAMVSIAYNALLQASGFIYVDGKGDNS
DotL_R.grylli FGNELSSKEELWFSNDDMRTHCLIFGSTGSGKTEALISLAYNALVQGSGFIYVDGKGDNS
DotL_L.pneumophila
α3 β5 TT TT α4
170 180 190 200 210 220
DotL_L.pneumophila LYAKVFSMVRSMGREDDLLLINFMTGARDIVGPQEKRLSNTLNPFCQGSSSMLTQLVVSL
DotL_A.lusitana LFAKVFSMCRSMGREDDLLLINFMTGARDIVGAQEKRLSNTMNPFCNGSSSMLSQLVVSL
DotL_B.aquae LYAKVFGMVRRMGRDDDLLLINFMTGARDVIGAQKSRLSNTMNPFANGSSSMLTQLVVSL
DotL_C.burnetii LFAKVFSMVRSMGREDDLLLINFMTGAKDIIGPQEKRLSNTLNPFANGSSSMLSQLIVSM
DotL_R.grylli LFAKVFSMARSMGREDDILVVNFMTGARDIVGPQEKRLSNTMNPFANGSSSMLSQLVVSL
DotL_L.pneumophila
α5 α6 α7
230 240 250 260 270 280
DotL_L.pneumophila MGSSGQSSDGDMWKGRAIAFVEALMRLLVYMRDEGAILLDANTIRNYFDLQRLESIVIDK
DotL_A.lusitana MDQGTQSADGDMWKGRAIVFVEALMKVLVYMRDQGKILLDANTIRNYFELTRLEAIAMDK
DotL_B.aquae MDSGGGGDGDMWKGRAISFVEALMKILVGMRDECHILLDANLIRSYFMLEKLEQIVVDR
DotL_C.burnetii MDKAAGTPDGDMWKGRAIAFTEALMKILVAMRDACHILLDANTIRNYFHLPKLEAIVVDK
DotL_R.grylli MDASSNTPDGDMWKGRAIVFVEALMKLLVYIRDQGGILLDVNVVRNYFELSRVENIVVDK
DotL_L.pneumophila
TT TT α8 η1 α9
290 300 310 320 330 340
DotL_L.pneumophila VFPRDDQESVNIETIPKLVTDPLRNYLNTLPGYNKEKKGKQVSQVLEQHGFITMQLVRSF
DotL_A.lusitana MFPRDNQEPVSLAETPDLVLEPIRNYIINLPGYDRSKKGKQGSQVLEQHGFITMQLTRAF
DotL_B.aquae RFPLNERESIPLDALESAYLDPIDNYIYNLPGYNKAKKGNQAGEVREQHGFITMQLGRTF
DotL_C.burnetii LFLREGEEPINLENVPPTVIEPIVNYVLTLPGYNKEKKGKQVSQVLEQHGFITMQLVRVF
DotL_R.grylli LFPRDGQEPLSLMDVPDVVLEPITNYLINLPGYDKSKKGKQVSQVLEQHGFITMQLTRVF
DotL_L.pneumophila
TTT α10 β6 α11
350 360 370 380 390 400
DotL_L.pneumophila SSLADTYGHIIRTNLAEVDFKDVVLNRRILVVLLPALEKSPDELSNLGKIIVSSLKAMMA
DotL_A.lusitana GSLADTYGHIIRTNLAEVDLKDVVLNRRILVVLLPALEKSPDELANLGKTIIASLKAMMA
DotL_B.aquae SSLADTYAHIVRTNLAEVDLKDVVLNRRILVVLLPALEKSPEELSNLGKIIIASLKAMMA
DotL_C.burnetii TSLADTYGHIMRTNLAEVDLRDVVLNRRILCVLLPALEKSPDELSNLGKVIVSTLKAMMA
DotL_R.grylli GSLADTYGHILRTNLAEVDLKDVVLNRRILLVLLPAFEKSPDELSNLGKVIIASLKAMLA

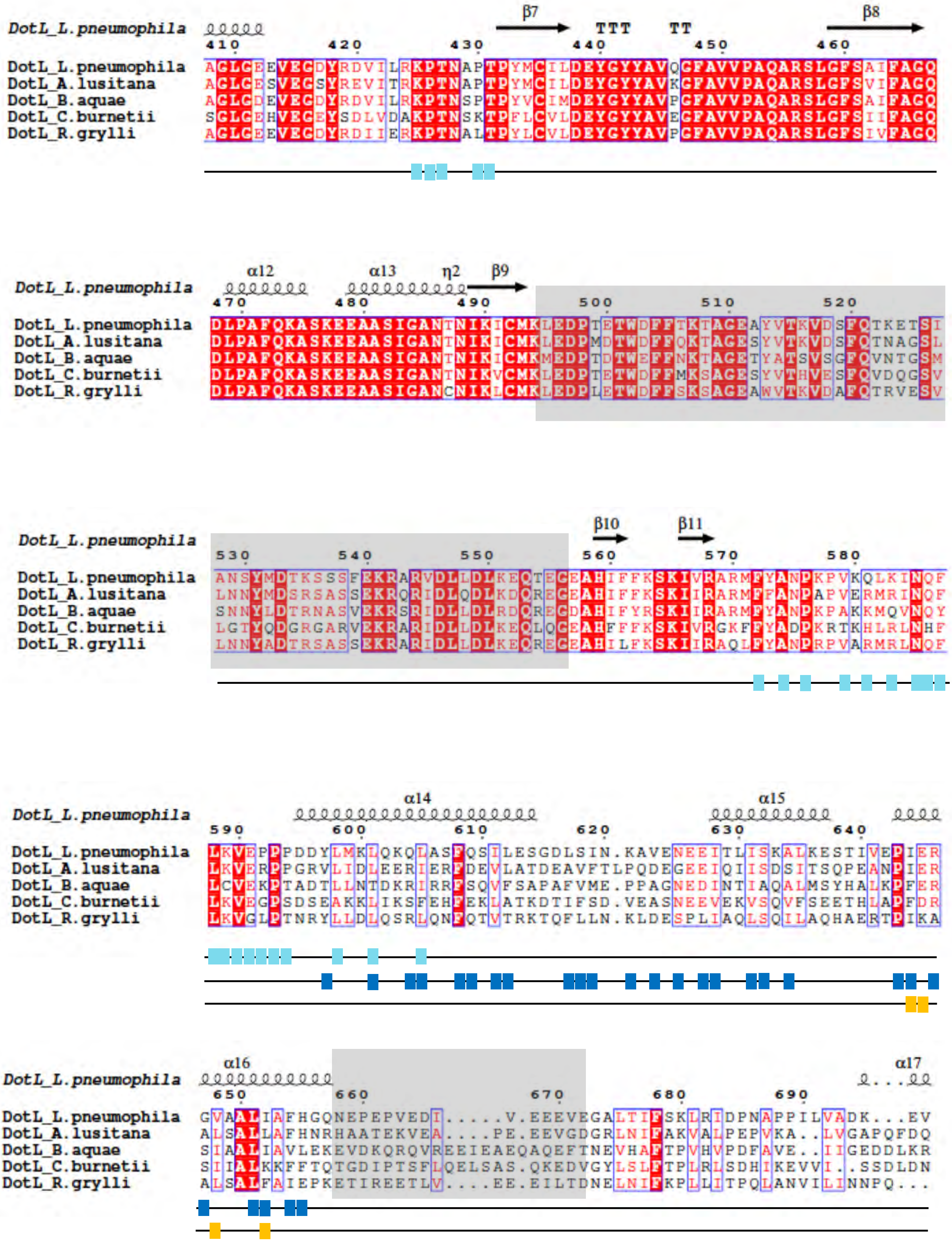

DotL_L.pneumophila
β7 TTT TT β8
410 420 430 440 450 460
DotL_L.pneumophila AGLGEEVEGDYRDVILRKPTNAPTPYMCILDEYGYYAVQGFAVVPAQARSLGFSAIFAGQ
DotL_A.lusitana AGLGESVEGSYREVITRKPTNAPTPYMCILDEYGYYAVKGFAVVPAQARSLGFSVIFAGQ
DotL_B.aquae AGLGDEVEGDYRDVILRKPTNSPTPYVCIMDEYGYYAVPGFAVVPAQARSLGFSAIFAGQ
DotL_C.burnetii SGLGEHVEGEYSDLVDAKPTNSKTPFLCVLDEYGYYAVEGFAVVPAQARSLGFSIIFAGQ
DotL_R.grylli AGLGEEVEGDYRDIIERKPTNALTPYLCVLDEYGYYAVPGFAVVPAQARSLGFSIVFAGQ
DotL_L.pneumophila
α12 α13 η2 β9
470 480 490 500 510 520
DotL_L.pneumophila DLPAFQKASKEEAASIGANTNIKICMKLEDPTETWDFFTKTAGEAYVTKVDSFQTKETSI
DotL_A.lusitana DLPAFQKASKEEAASIGANTNIKICMKLEDPMDTWDFFQKTAGESYVTKVDSFQTNAGSL
DotL_B.aquae DLPAFQKASKEEAASIGANTNIKICMKMEDPTDTWEFFNKTAGETYATSVSGFQVNTGSM
DotL_C.burnetii DLPAFQKASKEEAASIGANTNIKVCMKLEDPTETWDFFMKSAGESYVTHVESFQVDQGSV
DotL_R.grylli DLPAFQKASKEEAASIGANCNIKLCMKLEDPLETWDFFSKSAGEAWVTKVDAFQTRVESV
DotL_L.pneumophila
β10 β11
530 540 550 560 570 580
DotL_L.pneumophila ANSYMDTKSSSFEKRARVDLLDLKEQTEGEAHIFFKSKIVRARMFYANPKPVKQLKINQF
DotL_A.lusitana LNNYMDSRSASSEKRQRIDLQDLKDQREGEAHIFFKSKIIRARMFFANPAPVERMRINQF
DotL_B.aquae SNNYLDTRNASVEKRSRIDLLDLRDQREGDAHIFYRSKIIRARMFYANPKPAKKMQVNQY
DotL_C.burnetii LGTYQDGRGARVEKRARIDLLDLKEQLQGEAHFFFKSKIVRGKFFYADPKRTKHLRLNHF
DotL_R.grylli LNNYADTRSASSEKRARIDLLDLKEQREGEAHILFKSKIIRAQLFYANPRPVARMRLNQF
DotL_L.pneumophila
α14 α15
590 600 610 620 630 640
DotL_L.pneumophila LKVEPPPDDYLMKLQKQLASFQSILESGDLSIN.KAVENEEITLISKALKESTIVEPIER
DotL_A.lusitana LKVERPPGRVLIDLEERIERFDEVLATDEAVFTLPQDEGEEIQIISDSITSQPEANPIER
DotL_B.aquae LCVEKPTADTLLNTDKRIRRFSQVFSAPAFVME.PPAGNEDINTIAQALMSYHALKPFER
DotL_C.burnetii LKVEGPSDSEAKKLIKSFEHFEKLATKDTIFSD.VEASNEEVEKVSQVFSEETHLAPFDR
DotL_R.grylli LKVGLPTNRYLLDLQSRLQNFQTVTRKTQFLLN.KLDESPLIAQLSQILAQHAERTPIKA
DotL_L.pneumophila
α16 α17
650 660 670 680 690
DotL_L.pneumophila GVAALIAFHGQNEPEPVEDI.....V.EEEVEGALTIFSKLRIDPNAPPILVADK...EV
DotL_A.lusitana ALSALLAFHNRHAATEKVEA....PE.EEVGDGRLNIFAKVALPEPVKA..LVGAPQFDQ
DotL_B.aquae SIAALIAVLEKEVDKQRQVREEIEAEQAQEFTNEVHAFTPVHVPDFAVE..IIGEDDLKR
DotL_C.burnetii SIIALKKFFTQTGDIPTSFLQELSAS.QKEDVGYLSLFTPLRLSDHIKEVVI..SSDLDN
DotL_R.grylli ALSALFAIEPKETIREETLV....EE.EILTDNELNIFKPLLITPQLANVILINNPQ...

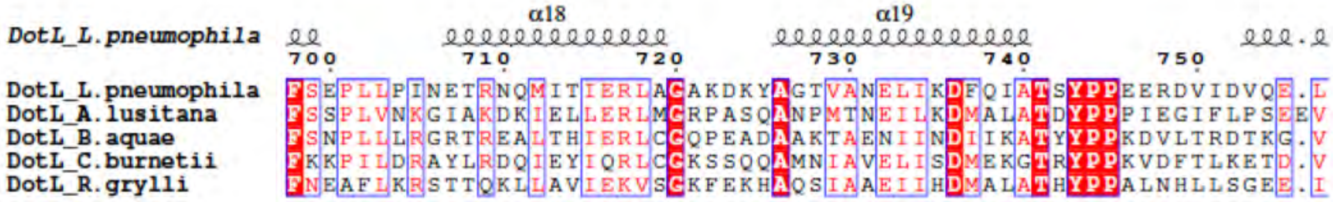

α18
α19
DotL_L.pneumophila
700
710
720
730
740
750
DotL_L.pneumophila FSEPLLPINETRNQMITIERLAGAKDKYAGTVANELIKDFQIATSYPPEERDVIDVQE.L
DotL_A.lusitana FSSPLVNKGIAKDKIELLERLMGRPASQANPMTNEILKDMALATDYPPPIEGIFLPSEEV
DotL_B.aquae FSNPLLLRGRTREALTHIERLCGQPEADAAKTAENIINDIIKATYYPPKDVLTRDTKG.V
DotL_C.burnetii FKKPILDRAYLRDQIEYIQRLCGKSSQQAMNIAVELISDMEKGTRYPPKVDFTLKETD.V
DotL_R.grylli FNEAFLKRSTTQKLLAVIEKVSGKFEKHAQSIAAEIIHDMALATHYPPALNHLLSGEE.I

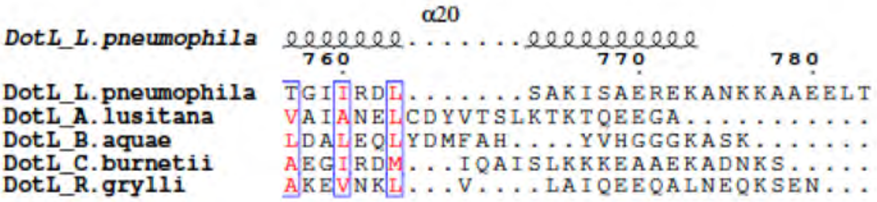

α20
DotL_L.pneumophila
760
770
780
DotL_L.pneumophila TGIIRDL.......SAKISAEREKANKKAAEELT
DotL_A.lusitana VAIANELCDYVTSLKTKTQEEGA...........
DotL_B.aquae LDALEQLYDMFAH....YVHGGGKASK.......
DotL_C.burnetii AEGIRDM...IQAISLKKKEAAEKADNKS.....
DotL_R.grylli AKEVNKL...V....LAIQEEQALNEQKSEN...

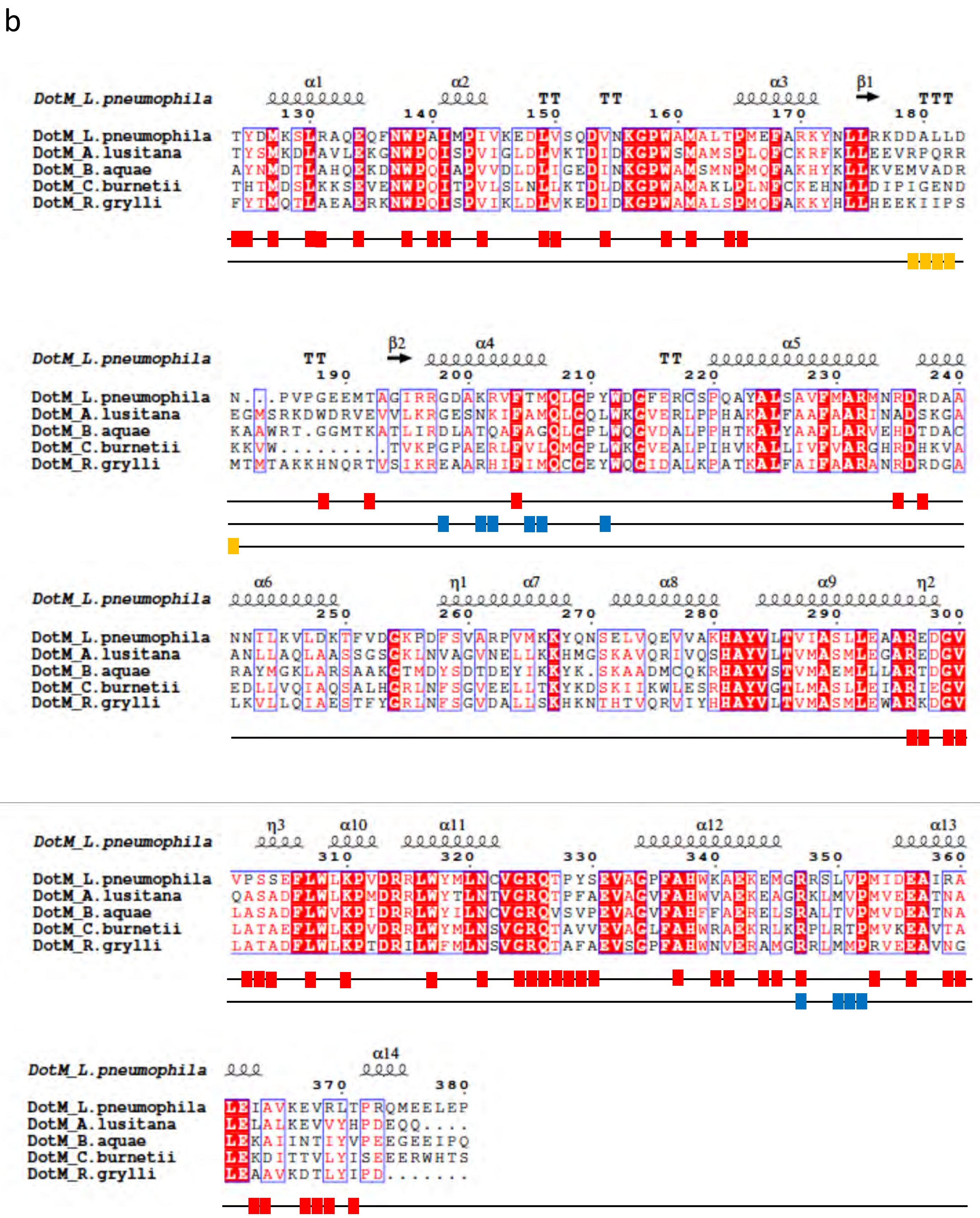
b
DotM_L.pneumophila
α1 α2 TT TT α3 β1 TTT
130 140 150 160 170 180
DotM_L.pneumophila TYDMKSLRAQEQFNWPAIMPIVKEDLVSQDVNKGPWAMALTPMEFARKYNLLRKDDALLD
DotM_A.lusitana TYSMKDLAVLEKGNWPQISPVIGLDLVKTDIDKGPWSMAMSPLQFCKRFKLLEEVRPQRR
DotM_B.aquae AYNMDTLAHQEKDNWPQIAPVVDLDLIGEDINKGPWAMSMNPMQFAKHYKLLKVEMVADR
DotM_C.burnetii THTMDSLKKSEVENWPQITPVLSLNLLKTDLDKGPWAMAKLPLNFCKEHNLLDIPIGEND
DotM_R.grylli FYTMQTLAEAERKNWPQISPVIKLDLVKEDIDKGPWAMALSPMQFAKKYHLLHEEKIIPS
DotM_L.pneumophila
TT β2 α4 TT α5
190 200 210 220 230 240
DotM_L.pneumophila N..PVPGEEMTAGIRRGDAKRVFTMQLGPYWDGFERCSPQAYALSAVFMARMNRDRDAA
DotM_A.lusitana EGMSRKDWDRVEVVLKRGESNKIFAMQLGQLWKGVERLPPHAKALFAAFAARINADSKGA
DotM_B.aquae KAAWRT.GGMTKATLIRDLATQAFAGQLGPLWQGVDALPPHTKALYAAFLARVEHDTDAC
DotM_C.burnetii KKVW.........TVKPGPAEERLFVLQMGPLWKGVEALPIHVKALLIVFVARGHRDHKVA
DotM_R.grylli MTMTAKKHNQRTVSIKREAARHIFIMQCGEYWQGIDALKPATKALFAIFAARANRDRDGA
DotM_L.pneumophila
α6 η1 α7 α8 α9 η2
250 260 270 280 290 300
DotM_L.pneumophila NNILKVLDKTFVDGKPDFSVARPVMKKYQNSELVQEVVAKHAYVLTVIASLLEAAREDGV
DotM_A.lusitana ANLLAQLAASSGSGKLNVAGVNELLKKHMGSKAVQRIVQSHAYVLTVMASMLEGAREDGV
DotM_B.aquae RAYMGKLARSAAKGTMDYSDTDEYIKKYK.SKAADMCQKRHAYVSTVMAEMLLLARTDGV
DotM_C.burnetii EDLLVQIAQSALHGRLNFSGVEELLTKYKDSKIIKWLESRHAYVGTLMASLLEIARIEGV
DotM_R.grylli LKVLLQIAESTFYGRLNFSGVDALLSKHKNTHTVQRVIYHHAYVLTVMASMLEWARKDGV
DotM_L.pneumophila
η3 α10 α11 α12 α13
310 320 330 340 350 360
DotM_L.pneumophila VPSSEFLWLKPVDRRLWYMLNCVGRQTPYSEVAGPFAHWKAEKEMGRRSLVPMIDEAIRA
DotM_A.lusitana QASADFLWLKPMDRRLWYTLNTVGRQTPFAEVAGVFAHWVAEKEAGRKLMVPMVEEATNA
DotM_B.aquae LASADFLWVKPIDRRLWYILNCVGRQVSVPEVAGVFAHFFAERELSRALTVPMVDEATNA
DotM_C.burnetii LATAEFLWLKPVDRRLWYMLNSVGRQTAVVEVAGLFAHWRAEKRLKRPLRTPMVKEAVTA
DotM_R.grylli LATADFLWLKPTDRILWFMLNSVGRQTAFAEVSGPFAHWNVERAMGRRLMMPRVEEAVNG
DotM_L.pneumophila
α14
370 380
DotM_L.pneumophila LEIAVKEVRLTPRQMEELEP
DotM_A.lusitana LELALKEVVYHPDEQQ....
DotM_B.aquae LEKAIINTIYVPEEGEEIPQ
DotM_C.burnetii LEKDITTVLYISEEERWHTS
DotM_R.grylli LEAAVKDTLYIPD.......

C

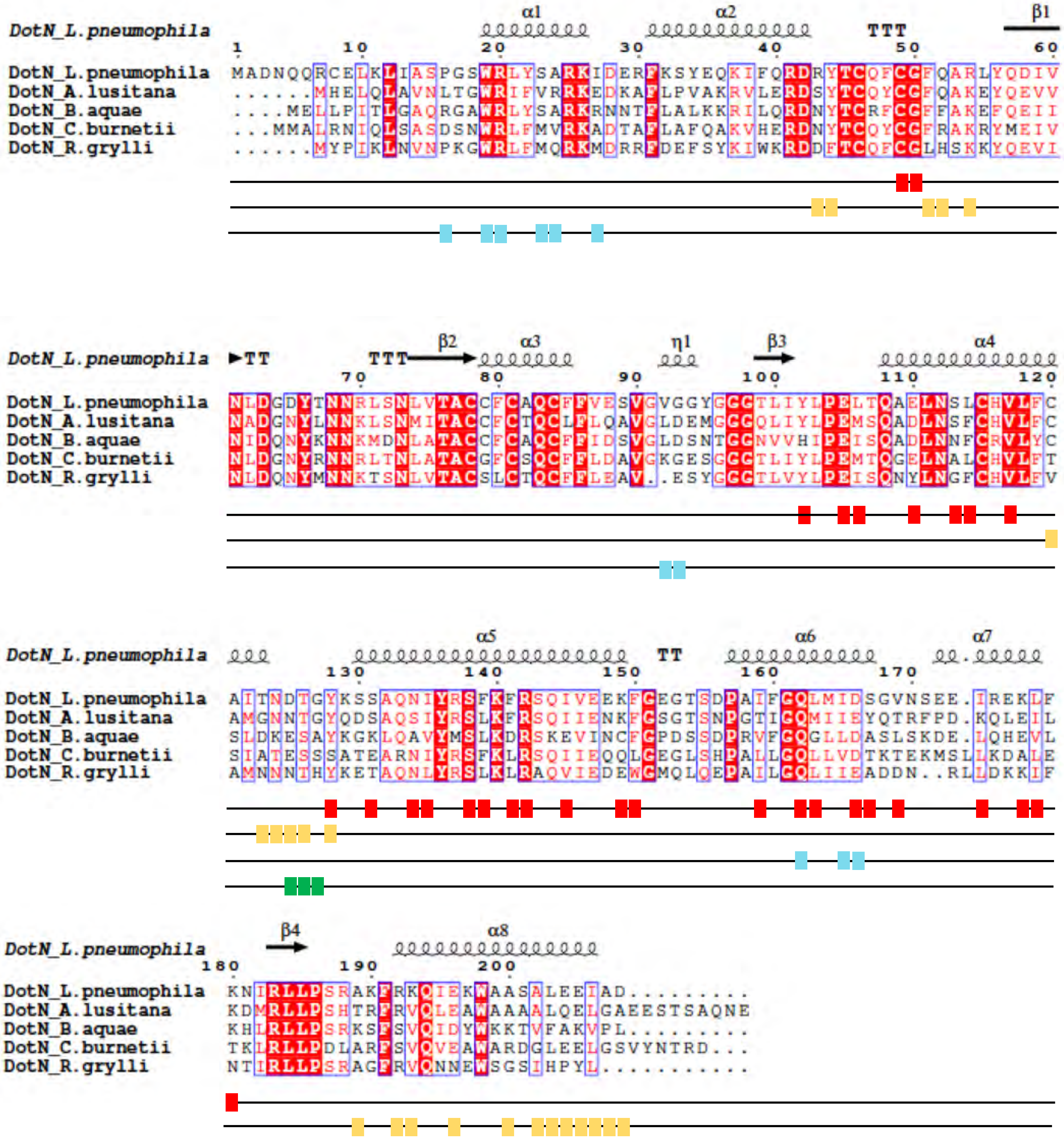
DotN_L.pneumophila
α1 α2 TTT β1
1 10 20 30 40 50 60
DotN_L.pneumophila MADNQQRCELKLIASPGSWRLYSARKIDERFKSYEQKIFQRDRYTCQFCGFQARLYQDIV
DotN_A.lusitana ......MHELQLAVNLTGWRIFVRRKEDKAFLPVAKRVLERDSYTCQYCGFQAKEYQEVV
DotN_B.aquae ....MELLPITLGAQRGAWRLYSARKRNNTFLALKKRILQRDNYTCRFCGFFAKEFQEII
DotN_C.burnetii ...MMALRNIQLSASDSNWRLFMVRKADTAFLAFQAKVHERDNYTCQYCGFRAKRYMEIV
DotN_R.grylli ......MYPIKLNVNPKGWRLFMQRKMDRRFDEFSYKIWKRDDFTCQFCGLHSKKYQEVI
DotN_L.pneumophila
TT TTT β2 α3 η1 β3 α4
70 80 90 100 110 120
DotN_L.pneumophila NLDGDYTNNRLSNLVTACCFCAQCFFVESVGVGGYGGGTLIYLPELTQAELNSLCHVLFC
DotN_A.lusitana NADGNYLNNKLSNMITACCFCTQCLFLQAVGLDEMGGGQLIYLPEMSQADLNSFCHVLFC
DotN_B.aquae NIDQNYKNNKMDNLATACCFCAQCFFIDSVGLDSNTGGNVVHIPEISQADLNNFCRVLYC
DotN_C.burnetii NLDGNYRNNRLTNLATACGFCSQCFFLDAVGKGESGGGTLIYLPEMTQGELNALCHVLFT
DotN_R.grylli NLDQNYMNNKTSNLVTACSLCTQCFFLEAV..ESYGGGTLVYLPEISQNYLNGFCHVLFV
DotN_L.pneumophila
α5 TT α6 α7
130 140 150 160 170
DotN_L.pneumophila AITNDTGYKSSAQNIYRSFKFRSQIVEEKFGEGTSDPAIFGQLMIDSGVNSEE.IREKLF
DotN_A.lusitana AMGNNTGYQDSAQSIYRSLKFRSQIIENKFGSGTSNPGTIGQMIIEYQTRFPD.KQLEIL
DotN_B.aquae SLDKESAYKGKLQAVYMSLKDRSKEVINCFGPDSSDPRVFGQGLLDASLSKDE.LQHEVL
DotN_C.burnetii SIATESSSATEARNIYRSFKLRSQIIEQQLGEGLSHPALLGQLLVDTKTEKMSLLKDALE
DotN_R.grylli AMNNNTHYKETAQNLYRSLKLRAQVIEDEWGMQLQEPAILGQLIIEADDN..RLLDKKIF
DotN_L.pneumophila
β4 α8
180 190 200
DotN_L.pneumophila KNIRLLPSRAKFRKQIEKWAASALEEIAD.........
DotN_A.lusitana KDMRLLPSHTRFRVQLEAWAAAALQELGAEESTSAQNE
DotN_B.aquae KHLRLLPSRKSFSVQIDYWKKTVFAKVPL.........
DotN_C.burnetii TKLRLLPDLARFSVQVEAWARDGLEELGSVYNTRD...
DotN_R.grylli NTIRLLPSRAGFRVQNNEWSGSIHPYL...........

d

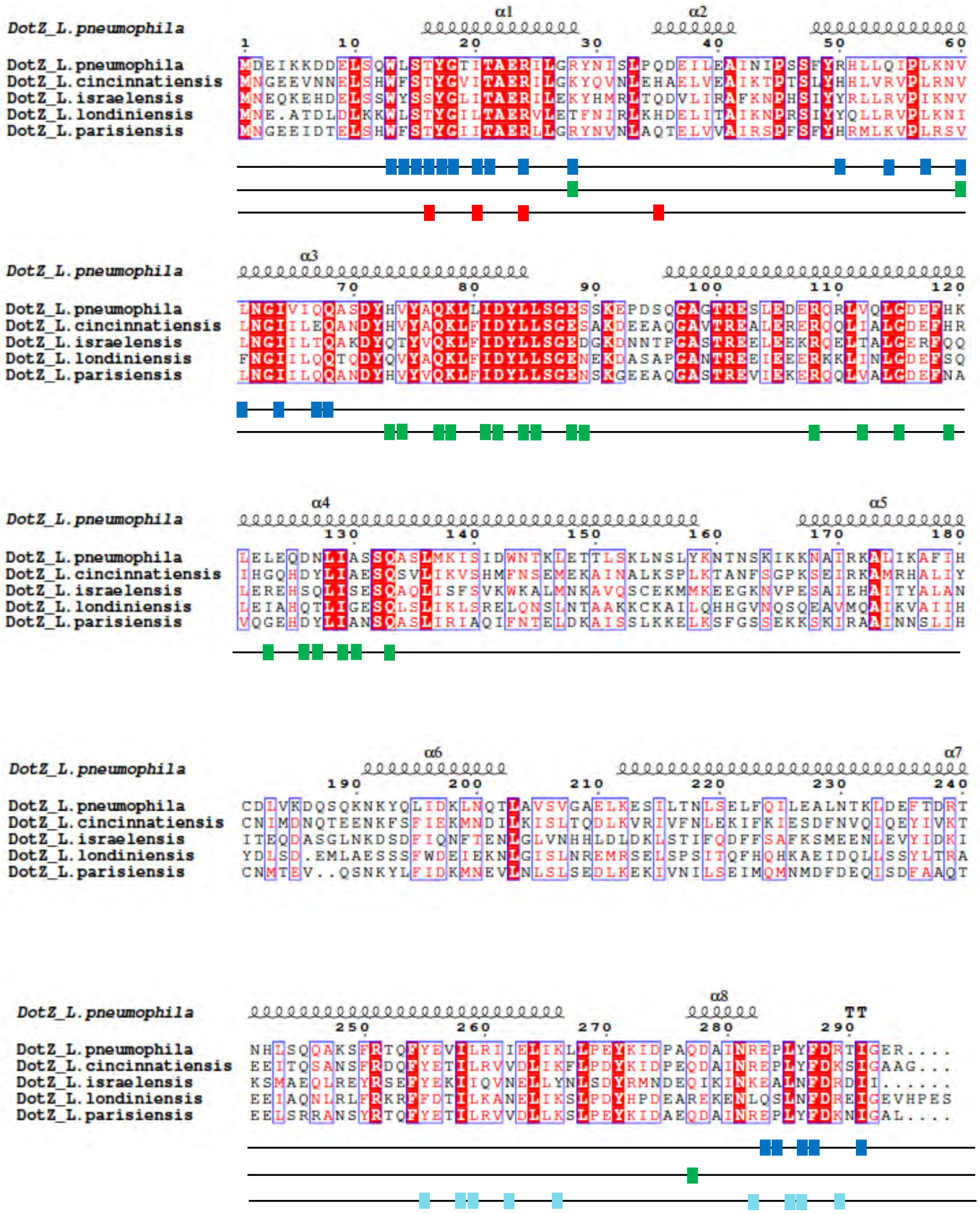
α1 α2
DotZ_L.pneumophila MDEIKKDDELSQWLSTYGTITAERILGRYNISLPQDEILEAINIPSSFYRHLLQIPLKNV
DotZ_L.cincinnatiensis MNGEEVNNELSHWFSTYGVITAERILGKYQVNLEHAELVEAIKTPTSLYHHLVRVPLRNV
DotZ_L.israelensis MNEQKEHDELSSWYSSYGLITAERILEKYHMRLTQDVLIRAFKNPHSIYYRLLRVPIKNV
DotZ_L.londiniensis MNE.ATDLDLKKWLSTYGILTAERVLETFNIRLKHDELITAIKNPRSIYYQLLRVPLKNI
DotZ_L.parisiensis MNGEEIDTELSHWFSTYGIITAERLLGRYNVNLAQTELVVAIRSPFSFYHRMLKVPLRSV
α3
DotZ_L.pneumophila LNGIVIQQASDYHVYAQKLLIDYLLSGESSKEPDSQGAGTRESLEDERQRLVQLGDEFHK
DotZ_L.cincinnatiensis LNGIILEQANDYHVYAQKLFIDYLLSGESAKDEEAQGAVTREALERERQQLIALGDEFHR
DotZ_L.israelensis LNGIILTQAKDYQTYVQKLFIDYLLSGEDGKDNNTPGASTREELEEKRQELTALGERFQQ
DotZ_L.londiniensis FNGIILQQTQDYQVYAQKLFIDYLLSGENEKDASAPGANTREEIEEERKKLINLGDEFSQ
DotZ_L.parisiensis LNGIILQQANDYHVYVQKLFIDYLLSGENSKGEEAQGASTREVIEKERQQLVALGDEFNA
α4 α5
DotZ_L.pneumophila LELEQDNLIASSQASLMKISIDWNTKLETTLSKLNSLYKNTNSKIKKNAIRKALIKAFIH
DotZ_L.cincinnatiensis IHGQHDYLIAESQSVLIKVSHMFNSEMEKAINALKSPLKTANFSGPKSEIRKAMRHALIY
DotZ_L.israelensis LEREHSQLISESQAQLISFSVKWKALMNKAVQSCEKMMKEEGKNVPESAIEHAITYALAN
DotZ_L.londiniensis LEIAHQTLIGESQLSLIKLSRELQNSLNTAAKKCKAILQHHGVNQSQEAVMQAIKVAIIH
DotZ_L.parisiensis VQGEHDYLIANSQASLIRIAQIFNTELDKAISSLKKELKSFGSSEKKSKIRAAINNSLIH
α6 α7
DotZ_L.pneumophila CDLVKDQSQKNKYQLIDKLNQTLAVSVGAELKESILTNLSELFQILEALNTKLDEFTDRT
DotZ_L.cincinnatiensis CNIMDNQTEENKFSFIEKMNDILKISLTQDLKVRIVFNLEKIFKIESDFNVQIQEYIVKT
DotZ_L.israelensis ITEQDASGLNKDSDFIQNFTENLGLVNHHLDLDKLSTIFQDFFSAFKSMEENLEVYIDKI
DotZ_L.londiniensis YDLSD.EMLAESSSFWDEIEKNLGISLNREMRSELSPSITQFHQHKAEIDQLLSSYLTRA
DotZ_L.parisiensis CNMTEV..QSNKYLFIDKMNEVLNLSLSEDLKEKIVNILSEIMQMNMDFDEQISDFAAQT
α8 TT
DotZ_L.pneumophila NHLSQQAKSFRTQFYEVILRIIELIKLLPEYKIDPAQDAINREPLYFDRTIGER....
DotZ_L.cincinnatiensis EEITQSANSFRDQFYETILRVVDLIKFLPDYKIDPEQDAINREPLYFDKSIGAAG...
DotZ_L.israelensis KSMAEQLREYRSEFYEKIIQVNELLYNLSDYRMNDEQIKINKEALNFDRDII......
DotZ_L.londiniensis EEIAQNLRLFRKRFFDTILKANELIKSLPDYHPDEAREKENLQSLNFDREIGEVHPES
DotZ_L.parisiensis EELSRRANSYRTQFYETILRVVDLLKSLPEYKIDAEQDAINREPLYFDKNIGAL....

e

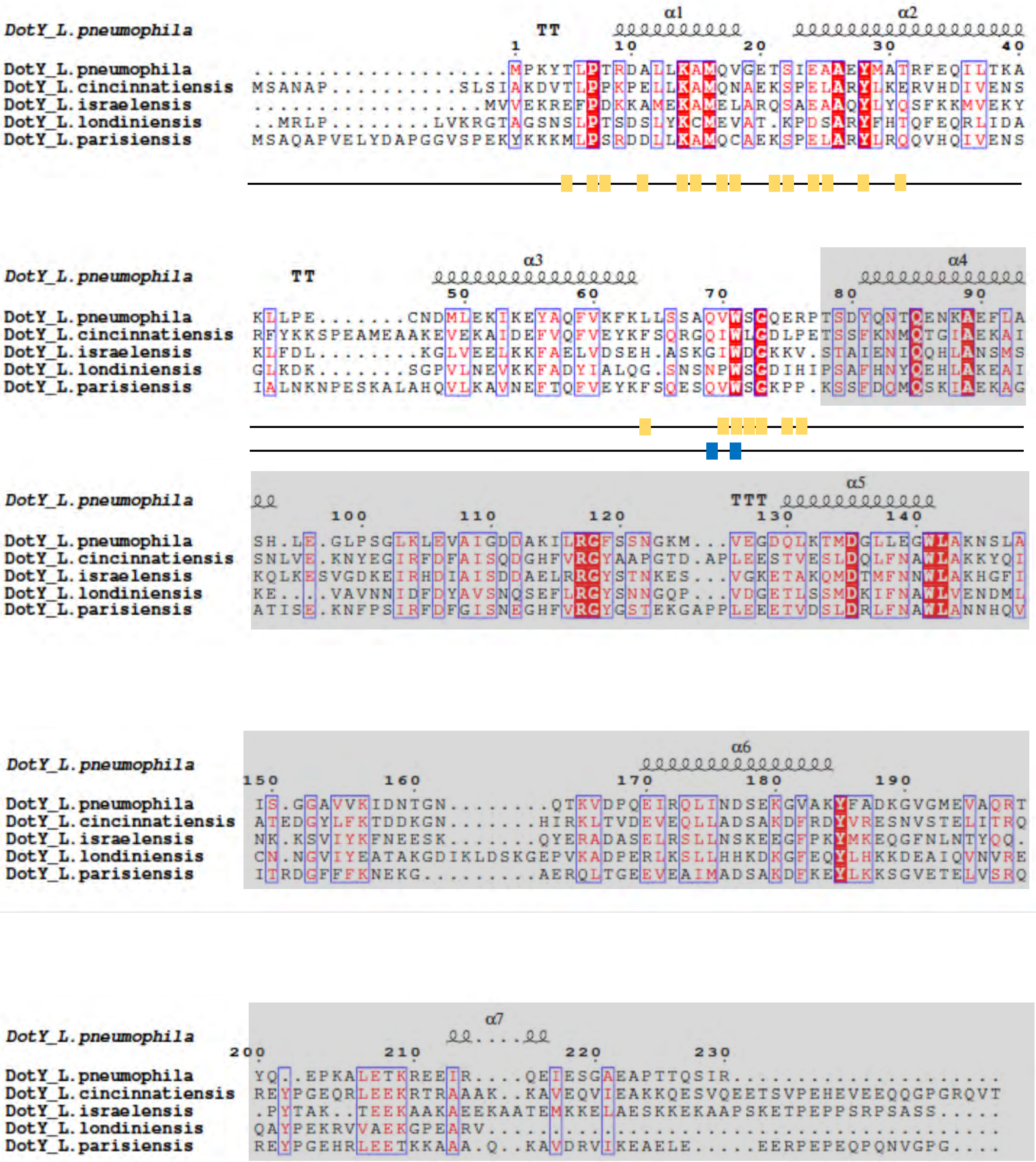

DotY_L.pneumophila
TT α1 α2
1 10 20 30 40
DotY_L.pneumophila ....................MPKYTLPTRDALLKAMQVGETSIEAAEYMATRFEQILTKA
DotY_L.cincinnatiensis MSANAP..........SLSIAKDVTLPPKPELLKAMQNAEKSPELARYLKERVHDIVENS
DotY_L.israelensis ................MVVEKREFPDKKAMEKAMELARQSAEAAQYLYQSFKKMVEKY
DotY_L.londiniensis ..MRLP........LVKRGTAGSNSLPTSDSLYKCMEVAT.KPDSARYFHTQFEQRLIDA
DotY_L.parisiensis MSAQAPVELYDAPGGVSPEKYKKKMLPSRDDLLKAMQCAEKSPELARYLRQQVHQIVENS
DotY_L.pneumophila
TT α3 α4
50 60 70 80 90
DotY_L.pneumophila KLLPE.......CNDMLEKIKEYAQFVKFKLLSSAQVWSGQERPTSDYQNTQENKAEFLA
DotY_L.cincinnatiensis RFYKKSPEAMEAAKEVEKAIDEFVQFVEYKFSQRGQIWLCDLPETSSFKNMQTGIAEKAI
DotY_L.israelensis KLFDL........KGLVEELKKFAELVDSEH.ASKGIWDCKKV.STAIENIQQHLANSMS
DotY_L.londiniensis GLKDK.......SGPVLNEVKKFADYIALQG.SNSNPWSCDIHIPSAFHNYQEHLAKEAI
DotY_L.parisiensis IALNKNPESKALAHQVLKAVNEFTQFVEYKFSQESQVWSCKPP.KSSFDQMQSKIAEKAG
DotY_L.pneumophila
TTT α5
100 110 120 130 140
DotY_L.pneumophila SH.LE.GLPSGLKLEVAIGDDAKILRGFSSNGKM...VEGDQLKTMDGLLEGWLAKNSLA
DotY_L.cincinnatiensis SNLVE.KNYEGIRFDFAISQDGHFVRGYAAPGTD.APLEESTVESLDQLFNAWLAKKYQI
DotY_L.israelensis KQLKESVGDKEIRHDIAISDDAELRRGYSTNKES...VGKETAKQMDTMFNNWLAKHGFI
DotY_L.londiniensis KE...VAVNNIDFDYAVSNQSEFLRGYSNNGQP...VDGETLSSMDKIFNAWLVENDML
DotY_L.parisiensis ATISE.KNFPSIRFDFGISNEGHFVRGYGSTEKGAPPLEEETVDSLDRLFNAWLANNHQV
DotY_L.pneumophila
α6
150 160 170 180 190
DotY_L.pneumophila IS.GGAVVKIDNTGN........QTKVDPQEIRQLINDSEKGVAKYFADKGVGMEVAQRT
DotY_L.cincinnatiensis ATEDGYLFKTDDKGN.......HIRKLTVDEVEQLLADSAKDFRDYVRESNVSTELITRQ
DotY_L.israelensis NK.KSVIYKFNEESK.......QYERADASELRSLLNSKEEGFPKYMKEQGFNLNTYQQ.
DotY_L.londiniensis CN.NGVIYEATAKGDIKLDSKGEPVKADPERLKSLLHHKDKGFEQYLHKKDEAIQVNVRE
DotY_L.parisiensis ITRDGFFFKNEKG.........AERQLTGEEVEAIMADSAKDFKEYLKKSGVETELVSRQ
DotY_L.pneumophila
α7
200 210 220 230
DotY_L.pneumophila YQ..EPKALETKREEIR....QEIESGAEAPTTQSIR....................
DotY_L.cincinnatiensis REYPGEQRLEEKRTRAAAK..KAVEQVIEAKKQESVQEETSVPEHEVEEQQGPGRQVT
DotY_L.israelensis .PYTAK..TEEKAAKAEEKAATEMKKELAESKKEKAAPSKETPEPPSRPSASS.....
DotY_L.londiniensis QAYPEKRVVAEKGPEARV..
DotY_L.parisiensis REYPGEHRLEETKKAAAA.Q..KAVDRVIKEAELE.....EERPEPEQPQNVGPG....

f

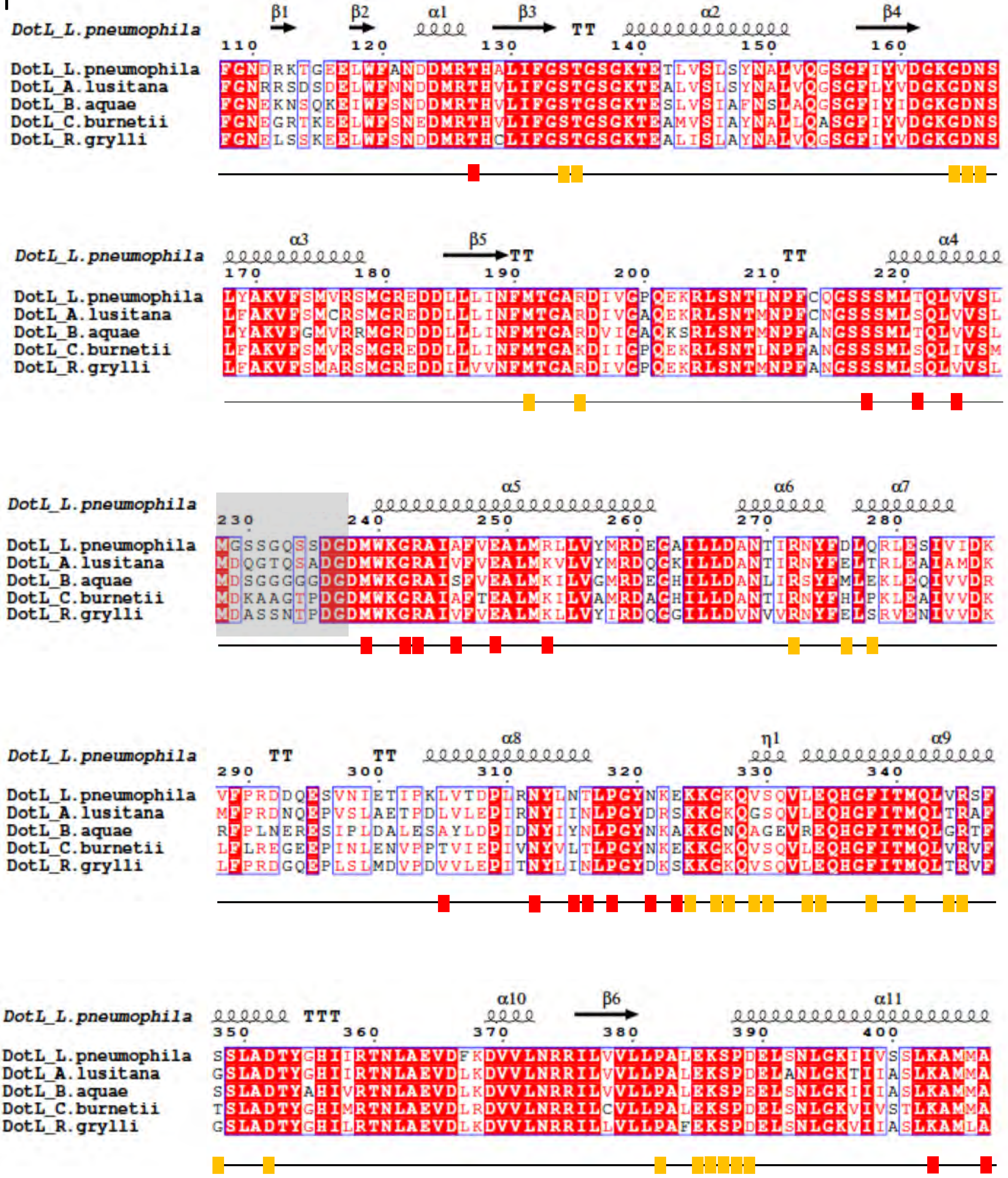
DotL_L.pneumophila
β1 β2 α1 β3 TT α2 β4
110 120 130 140 150 160
DotL_L.pneumophila FGNDRKTGEELWFANDDMRTHALIFGSTGSGKTETLVSLSYNALVQGSGFIYVDGKGDNS
DotL_A.lusitana FGNRRSDSDELWFNNDDMRTHVLIFGSTGSGKTEALVSLSYNALVQGSGFLYVDGKGDNS
DotL_B.aquae FGNEKNSQKEIWFSNDDMRTHVLIFGSTGSGKTESLVSIAFNSLAQGSGFIYIDGKGDNS
DotL_C.burnetii FGNEGRTKEELWFSNEDMRTHVLIFGSTGSGKTEAMVSIAYNALLQASGFIYVDGKGDNS
DotL_R.grylli FGNELSSKEELWFSNDDMRTHCLIFGSTGSGKTEALISLAYNALVQGSGFIYVDGKGDNS
DotL_L.pneumophila
α3 β5 TT TT α4
170 180 190 200 210 220
DotL_L.pneumophila LYAKVFSMVRSMGREDDLLLINFMTGARDIVGPQEKRLSNTLNPFCQGSSSMLTQLVVSL
DotL_A.lusitana LFAKVFSMCRSMGREDDLLLINFMTGARDIVGAQEKRLSNTMNPFCNGSSSMLSQLVVSL
DotL_B.aquae LYAKVFGMVRRMGRDDDLLLINFMTGARDVIGAQKSRLSNTMNPFANGSSSMLTQLVVSL
DotL_C.burnetii LFAKVFSMVRSMGREDDLLLINFMTGAKDIIGPQEKRLSNTLNPFANGSSSMLSQLIVSM
DotL_R.grylli LFAKVFSMARSMGREDDILVVNFMTGARDIVGPQEKRLSNTMNPFANGSSSMLSQLVVSL
DotL_L.pneumophila
α5 α6 α7
230 240 250 260 270 280
DotL_L.pneumophila MGSSGQSSDGDMWKGRAIAFVEALMRLLVYMRDEGAILLDANTIRNYFDLQRLESIVIDK
DotL_A.lusitana MDQGTQSADGDMWKGRAIVFVEALMKVLVYMRDQGKILLDANTIRNYFELTRLEAIAMDK
DotL_B.aquae MDSGGGGDGDMWKGRAISFVEALMKILVGMRDECHILLDANLIRSYFMLEKLEQIVVDR
DotL_C.burnetii MDKAAGTPDGDMWKGRAIAFTEALMKILVAMRDACHILLDANTIRNYFHLPKLEAIVVDK
DotL_R.grylli MDASSNTPDGDMWKGRAIVFVEALMKLLVYIRDQGGILLDVNVVRNYFELSRVENIVVDK
DotL_L.pneumophila
TT TT α8 η1 α9
290 300 310 320 330 340
DotL_L.pneumophila VFPRDDQESVNIETIPKLVTDPLRNYLNTLPGYNKEKKGKQVSQVLEQHGFITMQLVRSF
DotL_A.lusitana MFPRDNQEPVSLAETPDLVLEPIRNYIINLPGYDRSKKGKQGSQVLEQHGFITMQLTRAF
DotL_B.aquae RFPLNERESIPLDALESAYLDPIDNYIYNLPGYNKAKKGNQAGEVREQHGFITMQLGRTF
DotL_C.burnetii LFLREGEEPINLENVPPTVIEPIVNYVLTLPGYNKEKKGKQVSQVLEQHGFITMQLVRVF
DotL_R.grylli LFPRDGQEPLSLMDVPDVVLEPITNYLINLPGYDKSKKGKQVSQVLEQHGFITMQLTRVF
DotL_L.pneumophila
TTT α10 β6 α11
350 360 370 380 390 400
DotL_L.pneumophila SSLADTYGHIIRTNLAEVDFKDVVLNRRILVVLLPALEKSPDELSNLGKIIVSSLKAMMA
DotL_A.lusitana GSLADTYGHIIRTNLAEVDLKDVVLNRRILVVLLPALEKSPDELANLGKTIIASLKAMMA
DotL_B.aquae SSLADTYAHIVRTNLAEVDLKDVVLNRRILVVLLPALEKSPEELSNLGKIIIASLKAMMA
DotL_C.burnetii TSLADTYGHIMRTNLAEVDLRDVVLNRRILCVLLPALEKSPDELSNLGKVIVSTLKAMMA
DotL_R.grylli GSLADTYGHILRTNLAEVDLKDVVLNRRILLVLLPAFEKSPDELSNLGKVIIASLKAMLA

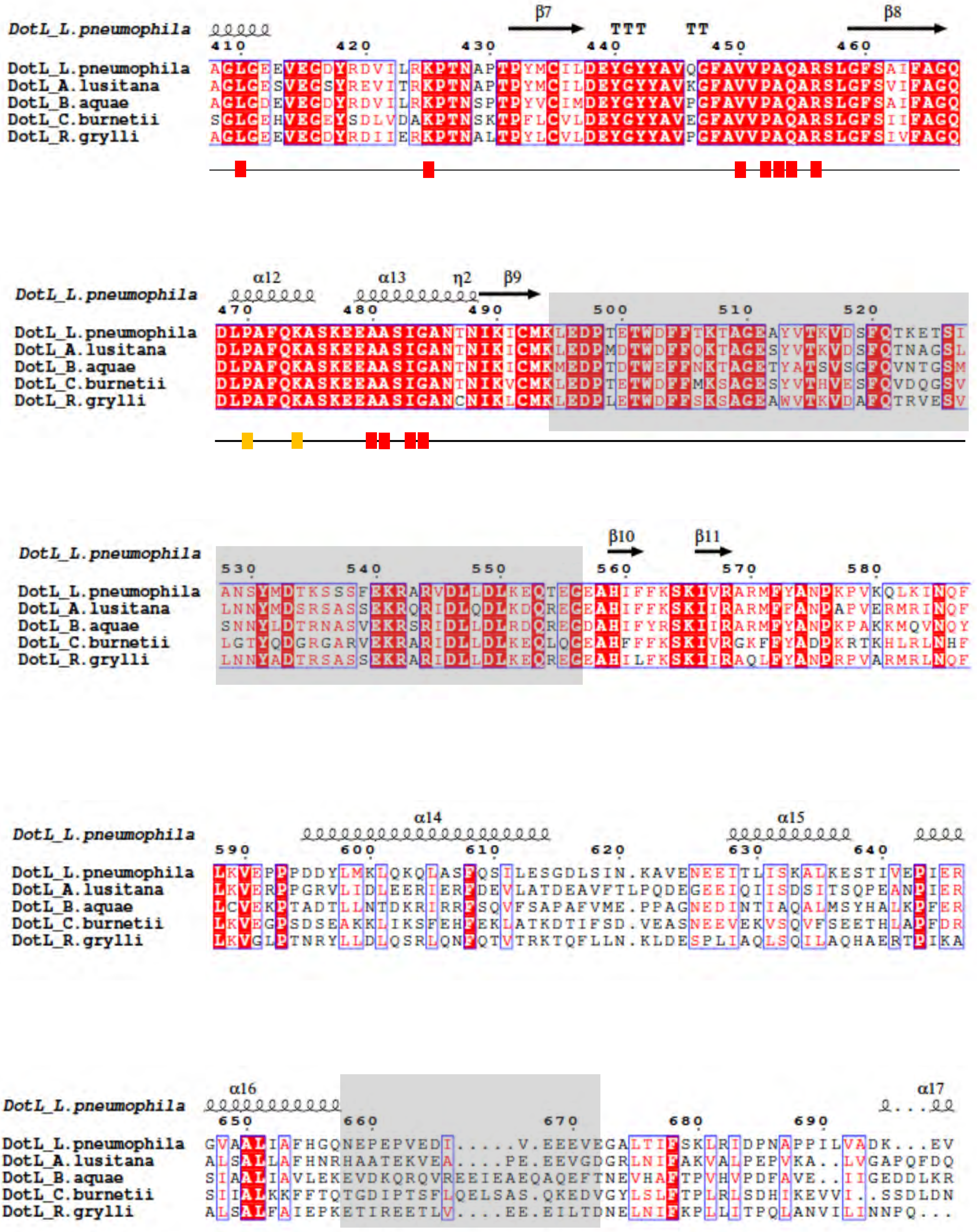

DotL_L.pneumophila
β7 TTT TT β8
410 420 430 440 450 460
DotL_L.pneumophila AGLGEEVEGDYRDVILRKPTNAPTPYMCILDEYGYYAVQGFAVVPAQARSLGFSAIFAGQ
DotL_A.lusitana AGLGESVEGSYREVITRKPTNAPTPYMCILDEYGYYAVKGFAVVPAQARSLGFSVIFAGQ
DotL_B.aquae AGLGDEVEGDYRDVILRKPTNSPTPYVCIMDEYGYYAVPGFAVVPAQARSLGFSAIFAGQ
DotL_C.burnetii SGLGEHVEGEYSDLVDAKPTNSKTPFLCVLDEYGYYAVEGFAVVPAQARSLGFSIIFAGQ
DotL_R.grylli AGLGEEVEGDYRDIIERKPTNALTPYLCVLDEYGYYAVPGFAVVPAQARSLGFSIVFAGQ
DotL_L.pneumophila
α12 α13 η2 β9
470 480 490 500 510 520
DotL_L.pneumophila DLPAFQKASKEEAASIGANTNIKICMKLEDPTETWDFFTKTAGEAYVTKVDSFQTKETSI
DotL_A.lusitana DLPAFQKASKEEAASIGANTNIKICMKLEDPMDTWDFFQKTAGESYVTKVDSFQTNAGSL
DotL_B.aquae DLPAFQKASKEEAASIGANTNIKICMKMEDPTDTWEFFNKTAGETYATSVSGFQVNTGSM
DotL_C.burnetii DLPAFQKASKEEAASIGANTNIKVCMKLEDPTETWDFFMKSAGESYVTHVESFQVDQGSV
DotL_R.grylli DLPAFQKASKEEAASIGANCNIKLCMKLEDPLETWDFFSKSAGEAWVTKVDAFQTRVESV
DotL_L.pneumophila
β10 β11
530 540 550 560 570 580
DotL_L.pneumophila ANSYMDTKSSSFEKRARVDLLDLKEQTEGEAHIFFKSKIVRARMFYANPKPVKQLKINQF
DotL_A.lusitana LNNYMDSRSASSEKRQRIDLQDLKDQREGEAHIFFKSKIIRARMFFANPAPVERMRINQF
DotL_B.aquae SNNYLDTRNASVEKRSRIDLLDLRDQREGDAHIFYRSKIIRARMFYANPKPAKKMQVNQY
DotL_C.burnetii LGTYQDGRGARVEKRARIDLLDLKEQLQGEAHFFFKSKIVRGKFFYADPKRTKHLRLNHF
DotL_R.grylli LNNYADTRSASSEKRARIDLLDLKEQREGEAHILFKSKIIRAQLFYANPRPVARMRLNQF
DotL_L.pneumophila
α14 α15
590 600 610 620 630 640
DotL_L.pneumophila LKVEPPPDDYLMKLQKQLASFQSILESGDLSIN.KAVENEEITLISKALKESTIVEPIER
DotL_A.lusitana LKVERPPGRVLIDLEERIERFDEVLATDEAVFTLPQDEGEEIQIISDSITSQPEANPIER
DotL_B.aquae LCVEKPTADTLLNTDKRIRRFSQVFSAPAFVME.PPAGNEDINTIAQALMSYHALKPFER
DotL_C.burnetii LKVEGPSDSEAKKLIKSFEHFEKLATKDTIFSD.VEASNEEVEKVSQVFSEETHLAPFDR
DotL_R.grylli LKVGLPTNRYLLDLQSRLQNFQTVTRKTQFLLN.KLDESPLIAQLSQILAQHAERTPIKA
DotL_L.pneumophila
α16 α17
650 660 670 680 690
DotL_L.pneumophila GVAALIAFHGQNEPEPVEDI.....V.EEEVEGALTIFSKLRIDPNAPPILVADK...EV
DotL_A.lusitana ALSALLAFHNRHAATEKVEA....PE.EEVGDGRLNIFAKVALPEPVKA..LVGAPQFDQ
DotL_B.aquae SIAALIAVLEKEVDKQRQVREEIEAEQAQEFTNEVHAFTPVHVPDFAVE..IIGEDDLKR
DotL_C.burnetii SIIALKKFFTQTGDIPTSFLQELSAS.QKEDVGYLSLFTPLRLSDHIKEVVI..SSDLDN
DotL_R.grylli ALSALFAIEPKETIREETLV....EE.EILTDNELNIFKPLLITPQLANVILINNPQ...

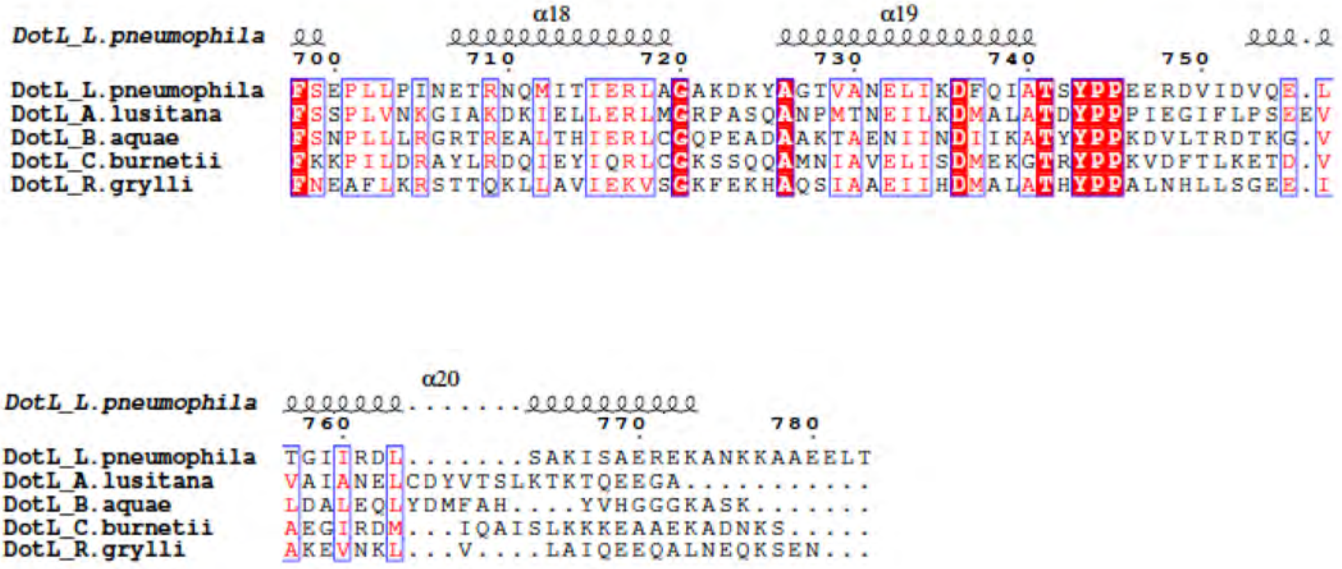
α18
α19
DotL_L.pneumophila
700
710
720
730
740
750
DotL_L.pneumophila FSEPLLPINETRNQMITIERLAGAKDKYAGTVANELIKDFQIATSYPPEERDVIDVQE.L
DotL_A.lusitana FSSPLVNKGIAKDKIELLERLMGRPASQANPMTNEILKDMALATDYPPPIEGIFLPSEEV
DotL_B.aquae FSNPLLLRGRTREALTHIERLCGQPEADAAKTAENIINDIIKATYYPPKDVLTRDTKG.V
DotL_C.burnetii FKKPILDRAYLRDQIEYIQRLCGKSSQQAMNIAVELISDMEKGTRYPPKVDFTLKETD.V
DotL_R.grylli FNEAFLKRSTTQKLLAVIEKVSGKFEKHAQSIAAEIIHDMALATHYPPALNHLLSGEE.I
α20
DotL_L.pneumophila
760
770
780
DotL_L.pneumophila TGIIRDL.......SAKISAEREKANKKAAEELT
DotL_A.lusitana VAIANELCDYVTSLKTKTQEEGA...........
DotL_B.aquae LDALEQLYDMFAH....YVHGGGKASK.......
DotL_C.burnetii AEGIRDM...IQAISLKKKEAAEKADNKS.....
DotL_R.grylli AKEVNKL...V....LAIQEEQALNEQKSEN...

Supplementary Figure 3. Sequence alignments and residues involved in interactions. Sequence alignments for DotL (a and f), DotM (b) and DotN (c) are reported between Legionellales species i.e. *Legionella pneumophila*, *Aquicella lusitana*, *Berkellia aquae*, *Coxiella burnetii*, and *Rickettsiella grylli*. For DotZ (d) and DotY (e), the sequence alignment is between *Legionella* species only. Residues in red boxes, or red or black letters are strictly conserved, similar or non-conserved, respectively. Secondary structural elements derived from the structure are shown above the sequence alignment with helices and strands represented by spirals and arrows, respectively. Alignment and secondary structure assignment were generated using Clustal Omega[8] and ESPrit[9]. Regions of DotL for which there was no electron density and therefore for which no model could be built are shown in grey. Interacting residues in the 8 interfaces observed in the T4CC hetero-pentameric complex are shown in colored boxes over lines under the sequence alignment in panels a-e. There is a different line for each interacting protein and the box under the interacting residue is color-coded according to the protein it interacts with (DotL, DotM, DotN, DotZ and DotY in red, cyan, blue, orange yellow, and green, respectively). For example, residues in DotL between 588 and 646 interact with 3 proteins, DotM, DotN, and DotZ and therefore 3 lines are show with boxes in cyan, blue, and orange yellow under the residues of DotL interacting with the corresponding proteins. Panel f reports on the residues of DotL which are predicted to be involved in interactions between adjacent subunits (color-coded red and orange) of the T4CC hexamer.

a

Protein 1	Protein 2	Interface (Å^2) and rank
DotL	DotM	3089 (1)
DotL	DotN	1756 (2)
DotL	DotZ	177 (7)
DotM	DotN	659 (5)
DotM	DotZ	355 (6)
DotN	DotZ	1517 (3)
DotN	DotY	101 (8)
DotZ	DotY	1396 (4)

b

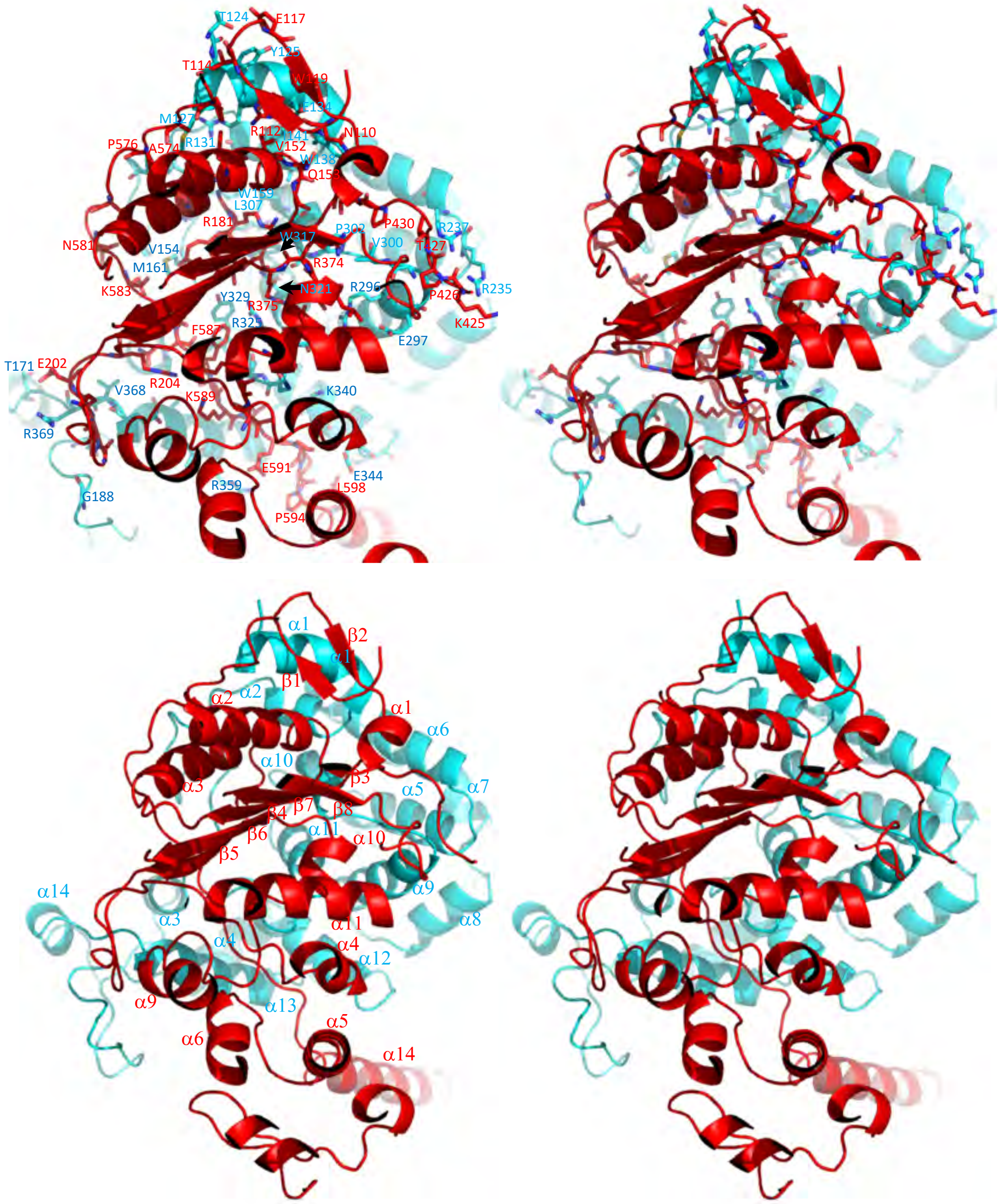

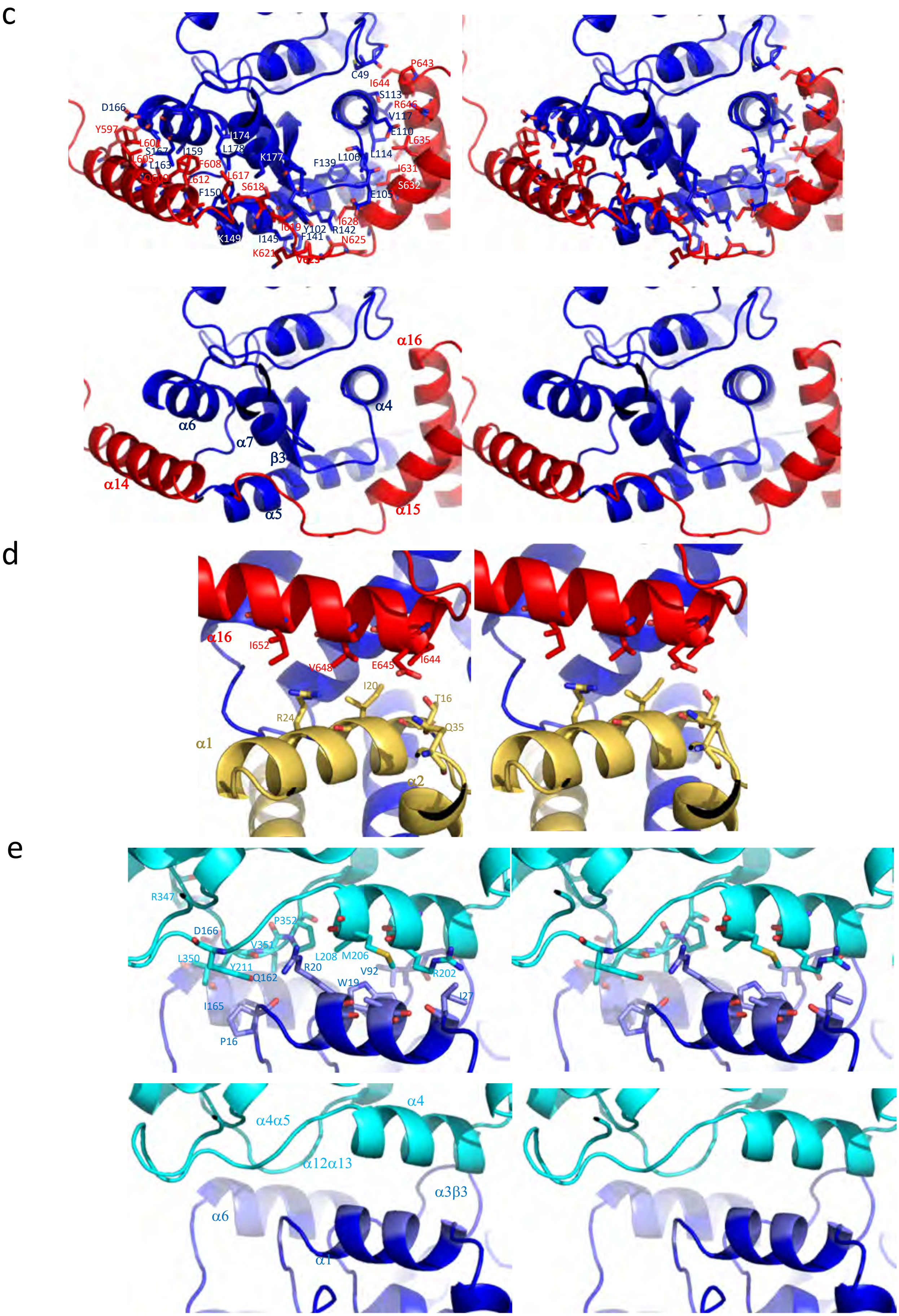
c
C49
P643
I644
S113
R646
D166
V117
Y597
E110
I174
L635
L601
L178
S167
I159
K177
L106
L114
F139
L605
L163
F608
I631
L617
L612
F150
S618
S632
E105
I628
I619
Y102
R142
K149
I145
F141
N625
K621
V623
α16
α4
α6
α7
β3
α14
α5
α15
d
α16
I652
V648
E645
I644
I20
T16
R24
Q35
α1
α2
e
R347
P352
D166
V351
L350
L208
M206
Y211
R20
V92
R202
Q162
W19
I27
I165
P16
α4α5
α4
α12α13
α3β3
α6
α1

f

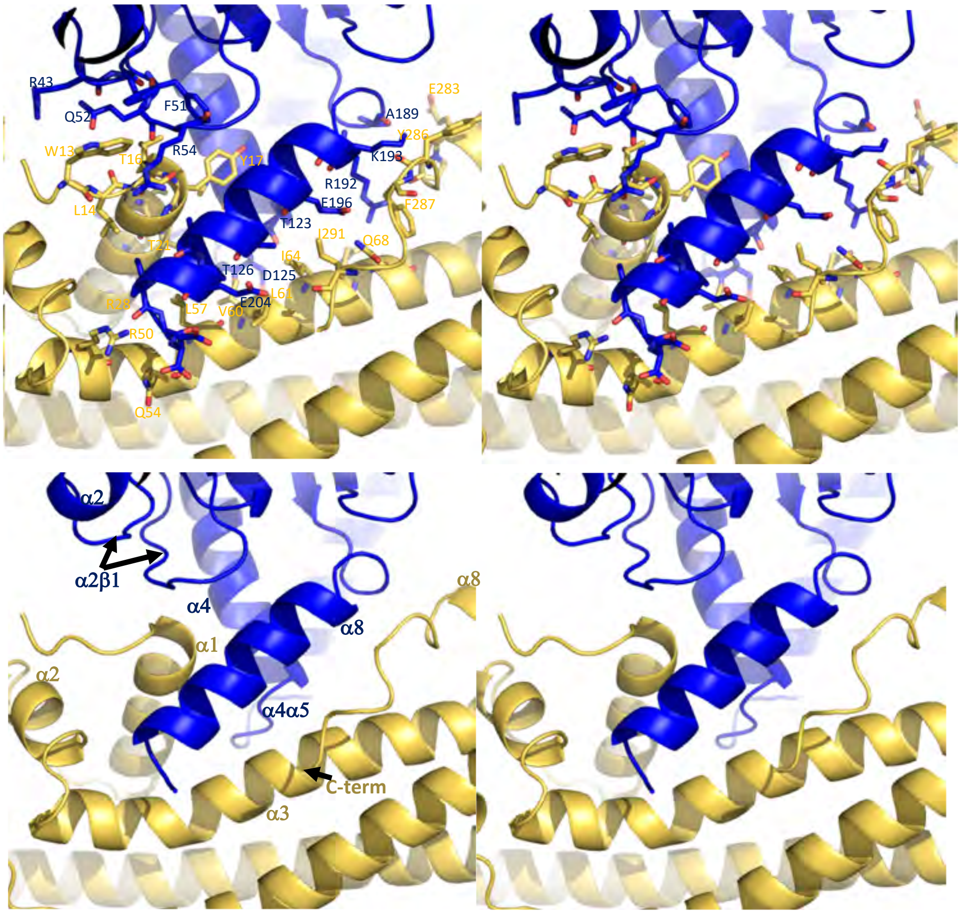
R43
Q52
F51
W13
T16
R54
Y17
A189
E283
Y286
K193
R192
E196
F287
L14
T123
I291
Q68
T24
I64
T126
D125
R28
E204
L61
L57
V60
R50
Q54
α2
α2β1
α4
α8
α1
α2
α4α5
C-term
α3

g

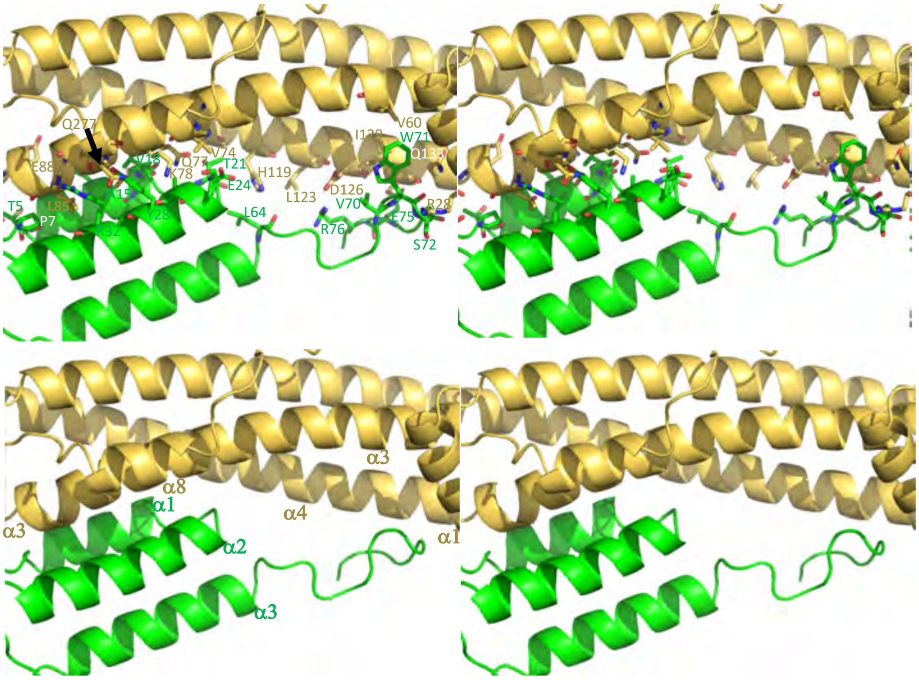
Q277
V60
I130
W71
E88
V18
V74
Q133
Q77
T21
K78
H119
E24
D126
T5
L85
L123
V70
R28
L64
P7
Y28
F75
R32
R76
S72
α3
α8
α1
α4
α3
α2
α1
α3

h

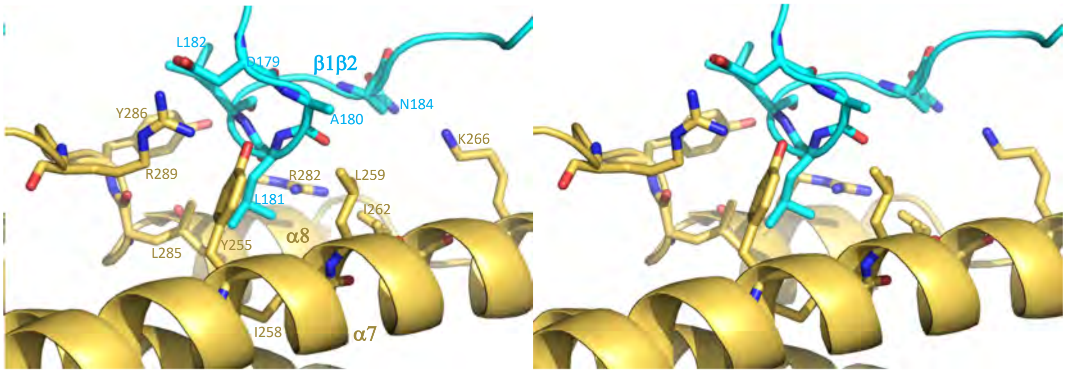
L182
D179
β1β2
N184
Y286
A180
K266
R289
R282
L259
L181
I262
α8
L285
Y255
I258
α7

i

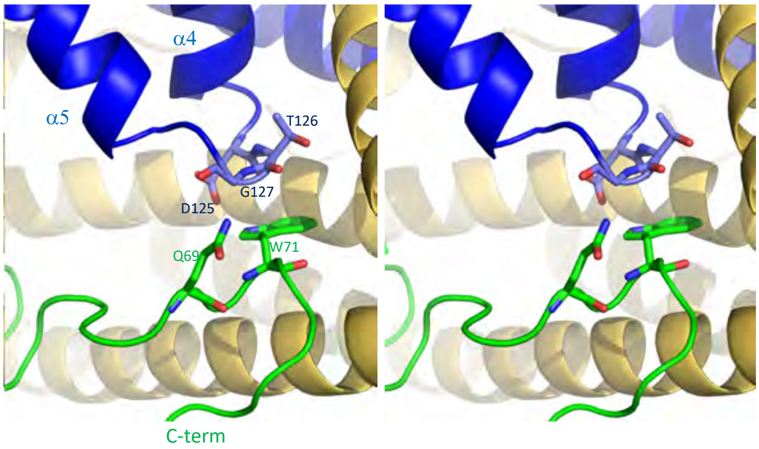
α4
α5
T126
G127
D125
Q69
W71
C-term

j

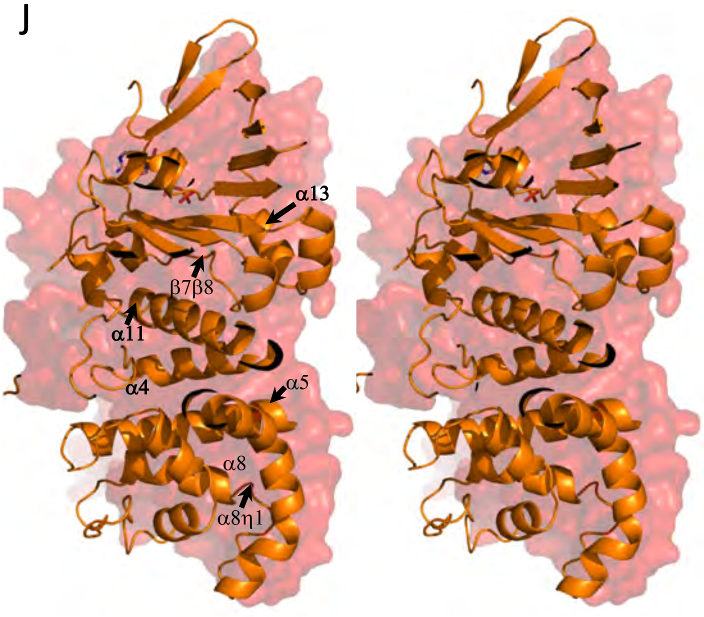
α13
β7β8
α11
α4
α5
α8
α8η1

k

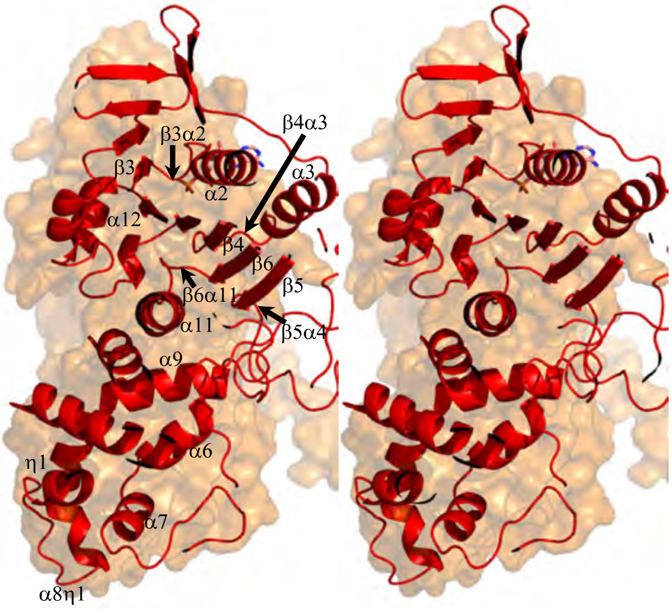
β3α2
β4α3
β3
α3
α2
α12
β4
β6
β5
β6α11
α11
β5α4
α9
α6
η1
α7
α8η1

Supplementary Figure 4. Residues and secondary structures involved in interactions between subunits of the hetero-pentameric complex (a-i) and between adjacent DotL subunits in the T4CC hexamer (j, k). Panels b-k show stereo figure diagrams of the regions of interest. **a**, Size of the 8 interfaces observed in the hetero-pentameric complex. Buried surface areas in each protein are reported in $Å^2$. **b**, Top: residues involved in DotL (red) - DotM (cyan) interactions; Bottom: secondary structure labelling involved in the interface. **c**, Residues (top) and secondary structures (bottom) involved in DotL (red) – DotN (blue) interactions. **d**, Residues and secondary structures involved in DotL (red) – DotZ (orange yellow) interactions. **e**, Residues (top) and secondary structures (bottom) involved in DotM (cyan) – DotN (blue) interactions. **f**, Residues (top) and secondary structures (bottom) involved in DotZ (orange yellow) – DotN (blue) interactions. **g**, Residues (top) and secondary structures (bottom) involved in DotY (green) – DotZ (orange yellow) interactions. **h**, Residues and secondary structures involved in DotM (cyan) – DotZ (orange yellow) interactions. **i**, Residues and secondary structures involved in DotY (green) – DotN (blue) interactions. **j and k**, Secondary structures involved in adjacent DotL-DotL interface shown in red and orange, respectively. In j, one subunit is shown in red surface and the adjacent one is in ribbon, while in k, it is the reverse.

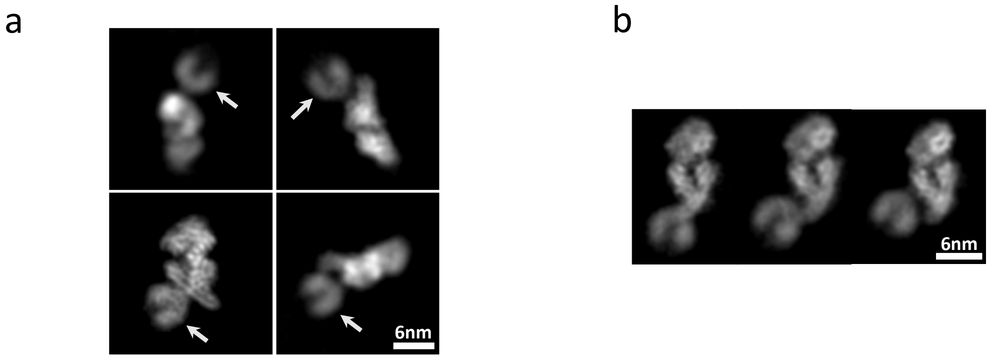

c

	Map 1	Map 2	Map 3	Map 4	Map 5	Map 6	Map 7
Nbr particles	2 513	2 579	3 828	13 181	1 034	4 775	3 877
CC IcmSW	0.94	0.93	0.94	0.94	0.94	0.93	0.94
CC T4CC core	0.90	0.89	0.92	0.91	0.93	0.93	0.93
σ level	2.0	2.0	1.8	1.7	2.0	1.4	1.2

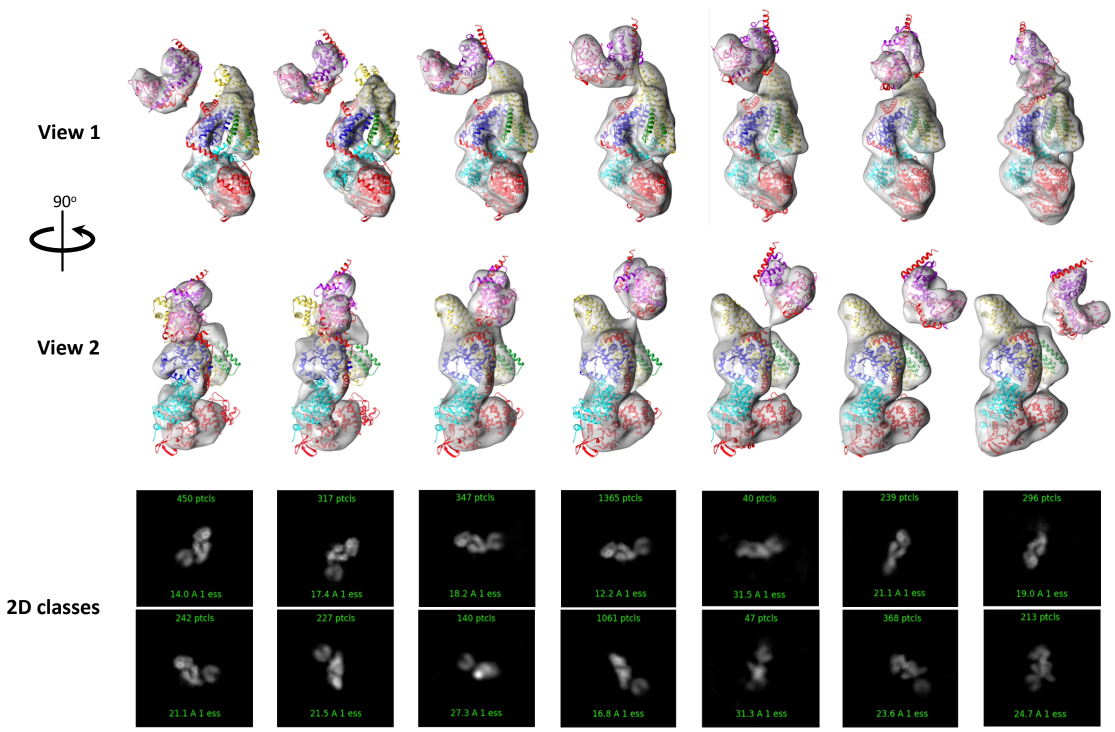

Supplementary Figure 5. Positional flexibility and trajectory of the IcmSW module. **a**, Four 2D class averages of the T4CC including IcmSW in various orientations. The U-shaped density is indicated in white arrows and was observed in at least 103,532 particles (see Methods). **b**, 2D class averages of the T4CC including IcmSW, showing 3 different locations of the IcmSW module. All averages show the T4CC hetero-pentameric core in the same orientation in order to illustrate the positional change of the IcmSW module. The number of particles used for these averages is indicated in panel **c**. **c**, Seven maps showing different positions of the IcmSW module were generated as described in Methods. This panel reports on the number of particles used to generate each map, the cross-correlation coefficient (CC)

for the fit of the IcmSW module and the T4CC hetero-pentameric core (labelled "T4CC core"), views of the maps and σ level at which they were contoured, and two examples of 2D class averages. The CCs were calculated using CHIMERA by comparing each experimental maps and a map calculated from either the IcmSW- $DotL_{672-783}$ model (PDB ID 5X1E) at 14 Å or the T4CC hetero-pentameric core structure (this work) at 8 Å.

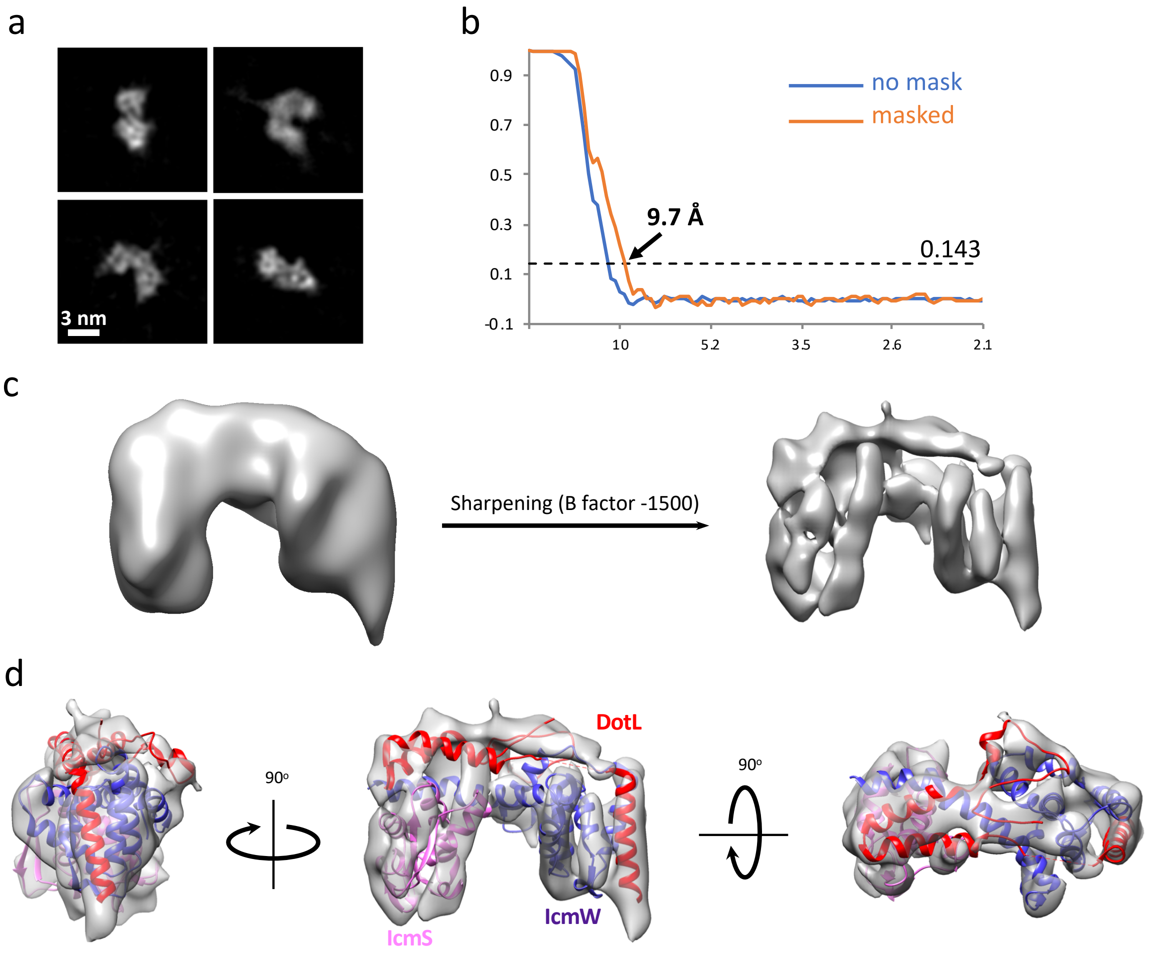

Supplementary Figure 6. 3D reconstruction of the U-shaped IcmSW density.
a, Examples of 2D class averages of views of the U-shaped density (see Methods section for the number of U-shape containing particles selected). 18,210 particles were used to generate these class averages. **b**, Resolution of the IcmSW module as derived from Fourier Shell Correlation (FSC) between independently refined half-maps. For the curve labelled "masked", the FSC was calculated using a mask. The dotted line represents the 0.143 FSC cut-off, which indicates a nominal resolution of 9.7 Å. **c**, Unsharpened and sharpened maps of the IcmSW module. The maps are contoured at 0.6 and 0.8 σ level, respectively. **d**, Orthogonal views of the U-shaped cryo EM map of the IcmSW module with the IcmSW-DotL$_{672\text{-}783}$ structure (PDB ID 5X1E) docked. The map is contoured at 0.8 σ level. The IcmSW-DotL$_{672\text{-}783}$ module is shown in ribbon representation, color-coded as in Fig.2. The cross-correlation coefficient, calculated using CHIMERA by comparing the experimental map and a map calculated from the IcmSW-DotL$_{672\text{-}783}$ structure (PDB ID 5X1E) at 9.7 Å resolution, is 0.92, indicating an excellent fit.

Supplementary References

1 Zuckman, D. M., Hung, J. B. & Roy, C. R. Pore-forming activity is not sufficient for *Legionella pneumophila* phagosome trafficking and intracellular growth. *Mol Microbiol* **32**, 990-1001 (1999).

2 Hubber, A. *et al.* The machinery at endoplasmic reticulum-plasma membrane contact sites contributes to spatial regulation of multiple *Legionella* effector proteins. *PLoS Pathog* **10**, e1004222 (2014).

3 Berger, K. H. & Isberg, R. R. Two distinct defects in intracellular growth complemented by a single genetic locus in *Legionella pneumophila*. *Mol Microbiol* **7**, 7-19 (1993).

4 Kubori, T. *et al.* Native structure of a type IV secretion system core complex essential for *Legionella* pathogenesis. *Proc Natl Acad Sci U S A* **111**, 11804-11809 (2014).

5 Nagai, H. *et al.* A C-terminal translocation signal required for Dot/Icm-dependent delivery of the *Legionella* RalF protein to host cells. *Proc Natl Acad Sci U S A* **102**, 826-831 (2005).

6 Meir, A., Chetrit, D., Liu, L., Roy, C. R. & Waksman, G. Legionella DotM structure reveals a role in effector recruiting to the Type 4B secretion system. *Nat Commun* **9**, 507 (2018).

7 Merriam, J. J., Mathur, R., Maxfield-Boumil, R. & Isberg, R. R. Analysis of the *Legionella pneumophila* fliI gene: intracellular growth of a defined mutant defective for flagellum biosynthesis. *Infect Immun* **65**, 2497-2501 (1997).

8 Madeira, F. *et al.* The EMBL-EBI search and sequence analysis tools APIs in 2019. *Nucleic Acids Res* **47**, W636-W641 (2019).

9 Robert, X. & Gouet, P. Deciphering key features in protein structures with the new ENDscript server. *Nucleic Acids Res* **42**, W320-324 (2014).

10 Bardill, J. P., Miller, J. L. & Vogel, J. P. IcmS-dependent translocation of SdeA into macrophages by the *Legionella pneumophila* type IV secretion system. *Mol Microbiol* **56**, 90-103 (2005).
