# Supplementary material for: Mechanism of effector capture and delivery by the type IV secretion system from Legionella pneumophila
Source: Nat Commun. 2020 Jun 8;11:2864. doi: 10.1038/s41467-020-16681-z (PMC7280309; doi:10.1038/s41467-020-16681-z)
Supplement: Supplementary file 3 — Reporting Summary [file 41467_2020_16681_MOESM3_ESM.pdf]

nature research

Corresponding author(s): Gabriel Waksman

Last updated by author(s): YYYY-MM-DD

Reporting Summary

Nature Research wishes to improve the reproducibility of the work that we publish. This form provides structure for consistency and transparency in reporting. For further information on Nature Research policies, see our Editorial Policies and the Editorial Policy Checklist.

Statistics

For all statistical analyses, confirm that the following items are present in the figure legend, table legend, main text, or Methods section.

n/a	Confirmed	
☐	☒	The exact sample size (n) for each experimental group/condition, given as a discrete number and unit of measurement
☐	☒	A statement on whether measurements were taken from distinct samples or whether the same sample was measured repeatedly
☐	☒	The statistical test(s) used AND whether they are one- or two-sided<br>*Only common tests should be described solely by name; describe more complex techniques in the Methods section.*
☒	☐	A description of all covariates tested
☐	☒	A description of any assumptions or corrections, such as tests of normality and adjustment for multiple comparisons
☐	☒	A full description of the statistical parameters including central tendency (e.g. means) or other basic estimates (e.g. regression coefficient) AND variation (e.g. standard deviation) or associated estimates of uncertainty (e.g. confidence intervals)
☐	☒	For null hypothesis testing, the test statistic (e.g. F, t, r) with confidence intervals, effect sizes, degrees of freedom and P value noted<br>*Give P values as exact values whenever suitable.*
☒	☐	For Bayesian analysis, information on the choice of priors and Markov chain Monte Carlo settings
☒	☐	For hierarchical and complex designs, identification of the appropriate level for tests and full reporting of outcomes
☒	☐	Estimates of effect sizes (e.g. Cohen's d, Pearson's r), indicating how they were calculated

Our web collection on statistics for biologists contains articles on many of the points above.

Software and code

Policy information about availability of computer code

Data collection	CryoEM data were collected by EPU software (Thermo Fisher, USA)
Data analysis	MotionCor2, CTFFIND 4.1, RELION 3.0, GAUTOMATCH 0.56, CRYOSPARC 0.6.5, PHENIX 1.14, i-TASSER, Microsoft Excel, UCSF Chimera 1.13.1, COOT 0.8.9.1, MOLPROBITY 4.4, CHIMERAX 0.91, PYMOL v2.3.2, Prism 8

For manuscripts utilizing custom algorithms or software that are central to the research but not yet described in published literature, software must be made available to editors and reviewers. We strongly encourage code deposition in a community repository (e.g. GitHub). See the Nature Research guidelines for submitting code & software for further information.

Data

Policy information about availability of data

All manuscripts must include a data availability statement. This statement should provide the following information, where applicable:

- Accession codes, unique identifiers, or web links for publicly available datasets
- A list of figures that have associated raw data
- A description of any restrictions on data availability

Entry codes for the EM density map and the atomic model of the hetero-pentameric T4CC core are EMD-10350 and PDB ID 6SZ9, respectively.
In vivo assays data is available in the source data file.

Field-specific reporting

Please select the one below that is the best fit for your research. If you are not sure, read the appropriate sections before making your selection.

☒ Life sciences ☐ Behavioural & social sciences ☐ Ecological, evolutionary & environmental sciences

For a reference copy of the document with all sections, see nature.com/documents/nr-reporting-summary-flat.pdf

Life sciences study design

All studies must disclose on these points even when the disclosure is negative.

Sample size	For complex purification, 3 Liters of LP grown to OD600-3.6-3.2 were harvested.<br>For CryoEM, 19,419 micrographs were collected over five data sets and 626,230 particles were used for reconstruction.<br>In vivo assays were conducted independently at least three times, with a biological triplicate for each bacterial strain (i.e., 3 wells/ mutant). For intracellular growth assay, 200,000 A. castellanii cells/well were infected with ~20,000 bacterial cells (MOI=0.1). Colonies were plated on CYE plates by a series of dilutions, and CFU were counted after 4-5 days. For Cya translocation assays, 100,000 CHO cells/well were infected with ~3,000,000 bacterial cells (MOI=30).
Data exclusions	CryoEM analysis involves the exclusion of data during processing based on image quality using criteria automatically determined by the software (for example, CFTFIND, Relion and CryoSparc). For in vivo assays, no data was excluded
Replication	For complex purification, bacteria growth and protein purifications were conducted at least 7 times yielding same complex profile (DotL, DotM, DotN, DotZ, DotY, IcmS, IcmW, LvgA). Proteins identities were verified by Mass Spectrometry a least twice from two independent experiments .Same complex profile was also observed in different detergents, different LP growth phase (OD=1), from two independently constructed colonies of the same strain, and from a deltaDotB background strain.<br>Initial CryoEM data collection and processing was conducted at least three times before five final high resolution data sets were collected.<br>For intracellular growth and Cya translocation assays, all experiments were conducted independently at least three times, each LP strain with a biological triplicate. The means of the resulting intracellular growth curves and cellular cAMP levels are presented with their standard deviations
Randomization	For EM data, this is not relevant. For In vivo assays, bacterial and Eukaryotic cells used in this study were randomly collected from a 2-days heavy patch (LP) or on-going cultivated cells (CHO and AC), each experiment with different batches.
Blinding	Particle picking and classification decisions done independently by A Meir and K. Mace were in agreement.<br>No blinding was performed in the in vivo tests.

Reporting for specific materials, systems and methods

We require information from authors about some types of materials, experimental systems and methods used in many studies. Here, indicate whether each material, system or method listed is relevant to your study. If you are not sure if a list item applies to your research, read the appropriate section before selecting a response.

Materials & experimental systems

n/a	Involved in the study
☒	☐ Antibodies
☐	☒ Eukaryotic cell lines
☒	☐ Palaeontology and archaeology
☒	☐ Animals and other organisms
☒	☐ Human research participants
☒	☐ Clinical data
☒	☐ Dual use research of concern

Methods

n/a	Involved in the study
☒	☐ ChIP-seq
☒	☐ Flow cytometry
☒	☐ MRI-based neuroimaging

Eukaryotic cell lines

Policy information about cell lines

Cell line source(s)	CHO FcγRII (Joiner 1990), Acanthamoeba castellanii (ATCC 30234)
Authentication	CHO cells were not authenticated. For AC, cells were acquired and authenticated by the American Type Culture Collection (ATCC)
Mycoplasma contamination	AC cells were confirmed negative for mycoplasma by the American Type Culture Collection (ATCC)
Commonly misidentified lines (See ICLAC register)	No commonly misidentified cells lines were used in this study.
